# Testing conditionality with Bourdieu's capital theory: How economic, social, and embodied cultural capital are associated with diet and physical activity in the Netherlands

**DOI:** 10.1016/j.ssmph.2023.101401

**Published:** 2023-04-10

**Authors:** Andrea L. Mudd, Joost Oude Groeniger, Michèlle Bal, Sanne E. Verra, Frank J. van Lenthe, Carlijn B.M. Kamphuis

**Affiliations:** a Department of Interdisciplinary Social Science- Social Policy and Public Health, Utrecht University, PO Box 80140, 3508, TC, Utrecht, the Netherlands; b Department of Public Health, Erasmus University Medical Centre, PO Box 2040, 3000, CA, Rotterdam, the Netherlands; c Department of Human Geography and Spatial Planning, Utrecht University, PO Box 80140, 3508, TC, Utrecht, the Netherlands

## Abstract

Although Bourdieu's capital theory emphasized that economic, social, and embodied cultural capital interact to shape health behavior, existing empirical research mainly considered separate associations of the three forms of capital. Our aim was to investigate if and how economic, social, and embodied cultural capital are conditional on each other in their associations with adults' diet and physical activity.

Cross-sectional, self-reported data from the 2014 GLOBE survey of 2812 adults aged between 25 and 75 years residing in Eindhoven, the Netherlands were used. Step-wise multiple logistic regression models included economic, social, and embodied cultural capital and adjustment for potential confounders. The models estimated odds ratios of main effects and two-way interactions of the forms of capital with fruit consumption, vegetable consumption, sports participation, and leisure time walking or cycling.

In the main effects models, embodied cultural capital was consistently positively associated with all outcomes. Social capital was positively associated with sports participation, fruit consumption, and vegetable consumption, and economic capital was positively associated with sports participation and vegetable consumption. In the two-way interaction models, having specific higher levels of both economic *and* social capital strengthened their positive association with sports participation. No other combinations of capital were conditional on each other.

Economic and social capital were conditional on each other in their association with sports participation, so interventions that provide both economic and social support may be especially effective for increasing this type of physical activity. As its association was strong with all outcomes but not conditional on other forms of capital, embodied cultural capital may operate distinctly from economic and social resources. Policy that takes differences in embodied cultural capital into account or changes to the environment that dampen the importance of embodied cultural resources for health behavior may help improve both diet and physical activity.

## Highlights

• Economic, social, and embodied cultural capital may interact to shape diet and PA.
• Economic and social capital interacted to boost sports participation only.
• Embodied cultural capital was positively related to all diet and PA measures.
• Embodied cultural capital was not conditional on social or economic capital.
• Policy should account for cultural capital or lower its relevance for diet and PA.

## 1 Background

Unhealthy diet and insufficient physical activity are two prevalent health behaviors linked to various chronic health conditions, such as heart disease and type 2 diabetes (Blair et al., 1996; Ford et al., 2011). The role of psychological determinants, including motivation, stress, and self-efficacy, in shaping diet and physical activity has often been emphasized in literature on the determinants of health behavior (Cortis et al., 2017; Jelsma et al., 2018; Lugones-Sanchez et al., 2021). Yet, it is crucial to look beyond individual responsibility and understand how factors at the structural level, the broader contexts and resources in society, influence health behavior (Diez Roux, 1998; Macintyre et al., 1993). More specifically, a different approach to understanding the drivers of health behavior is to consider how the economic, social, and cultural resources one has access to constrain available opportunities to be healthy by, for example, applying French sociologist Pierre Bourdieu's theory of capital (Short & Mollborn, 2015; Weaver et al., 2014). Investigating how different combinations of resources shape dietary behaviors and physical activity could inform the design of policies that help increase the uptake of healthier behaviors.

In his theory of capital, Bourdieu argued that one's social position grants access to different types of resources, specifically economic, social, and cultural capital (Bourdieu, 1986). Social position dictates the economic, social, and cultural resources one has at their disposal, and these resources can be leveraged towards certain lifestyle practices (Frohlich & Abel, 2014). Understanding how these lifestyle practices are embedded in social class and the way resources are distributed across society may help explain the persistent prevalence of unhealthy behavior, including unhealthy diet and insufficient physical activity (Collyer, 2015). In this study, we apply Bourdieu's definitions of economic, social, and cultural capital (Bourdieu, 1986; Bourdieu & Wacquant, 1992). Economic capital refers to material resources that are immediately and directly convertible into money. Social capital is the size and quality of the network of connections a person has. Cultural capital, broadly defined as social and informational resources for action, exists in three states: *objectified* (access to cultural goods like books and art), *institutionalized* (e.g., academic degrees and professional titles), and *embodied* (e.g., knowledge, values, and skills) cultural capital. Abel previously theorized that *embodied* cultural capital is the type of cultural capital that can most directly influence health behavior (Abel, 2008). Unlike objectified and institutionalized cultural capital, embodied cultural capital cannot be directly conveyed or transferred to someone else. Embodied cultural capital is a representation of internalized cultural capital developed through the long-lasting process of socialization (Kamphuis, Jansen, Mackenbach, & Van Lenthe, 2015; Lareau & Weininger, 2003). This makes embodied cultural capital a resource integral to a person's capacity for agency (the ability to act on one's will) that can be activated towards health behavior.

The forms of capital are theorized to interact in multiple ways in their influence on health behavior (Bourdieu, 1986). Bourdieu wrote about capital conversion, accumulation, and transmission, and, more recently, Abel and Frohlich proposed “conditionality” as an important way the three forms of capital may interact (Abel & Frohlich, 2012; Bourdieu, 1986). A form of capital is conditional on another if having a specific level of that form of capital can boost or limit the use and acquisition of the other (Abel & Frohlich, 2012). For example, more income (economic capital) affords the opportunity to join a sports club, but people may be more likely to join and go to the sports club if they also value a healthy lifestyle (embodied cultural capital) or have peers to work out with (social capital); in this example, embodied cultural capital and social capital boost the use of economic capital towards healthier behavior.

Empirical research into how the three forms of capital impact diet and physical activity has largely focused on the separate associations of each form of capital (Kamphuis, Oude Groeniger, & van Lenthe, 2018; Wilson, 2002; Xu & Jiang, 2020). Findings from these studies suggest that social and cultural capital are more important drivers of diet and physical activity than economic capital. The few empirical studies that have explored interrelations between the forms of capital with regards to health and health behavior found mixed evidence of interaction effects (De Clercq et al., 2016; Pinxten & Lievens, 2014; Veenstra & Patterson, 2012). We aim to contribute to the literature by explicitly investigating conditionality between the three forms of capital, by investigating these interrelations for both diet and physical activity, and by formulating specific, theory-driven hypotheses about how we expect the forms of capital to be conditional on each other in their associations with diet and physical activity.

The primary aim of this study is to investigate how economic, social, and embodied cultural capital are conditional on each other in their influence on healthy diet and physical activity for adults in the social and geographical context of the Netherlands. In order to investigate these interrelations, we will also study the separate associations of the three forms of capital with diet and physical activity. We expect that those with a higher level of each individual form of capital are more likely to consume a healthier diet and engage in more physical activity, though we expect that economic capital may be less relevant for these behaviors, in line with the relationships established in the literature (Kamphuis, Oude Groeniger, & van Lenthe, 2018; Wilson, 2002; Xu & Jiang, 2020).

Regarding potential conditionalities between combinations of the forms of capital, we expect that having a higher level of a given form of capital will boost the influence of another on diet and physical activity. First, we expect that economic and embodied cultural capital are conditional on each other. Options for participating in certain health behaviors are dictated by the amount of economic resources one has access to (e.g., the ability to purchase a gym membership or fresh fruits and vegetables). Within these available options, one's actual health lifestyle may be influenced by norms, perceptions, and knowledge (Abel, 2008). This means that those with plentiful economic resources are expected to be more likely to have a healthy diet and engage in recommended levels of physical activity if they value these particular health-related behaviors and have the skills and knowledge to execute them (put otherwise, if they have higher embodied cultural capital). Second, we expect that social and embodied cultural capital are conditional on each other. Within a social network that has the potential to increase healthy behavior, having stronger shared values and operational skills for health promotion could help promote each social network member's participation in healthy activities (Abel, 2008). Similarly, knowledge and values may interact with one's social network and influences to inform decisions to behave a certain way (Flay & Petraitis, 1994). Specific to our study, having a strong, supportive social network may be more likely to benefit diet and physical activity when paired with an embodied repertoire to pursue a healthy lifestyle. Finally, we expect that economic and social capital are conditional on each other. This conditionality was observed in qualitative interviews with diabetes patients, where low economic resources coupled with limited chances for meaningful social engagement reduced motivation and capabilities to be healthy (Weaver et al., 2014). In the context of diet and physical activity, putting economic resources towards a healthier diet and physical activity may be more likely when supported by a social network that encourages a healthy lifestyle. By deepening understanding of how these types of resources are conditional on each other, this study can help inform policy that aims to provide those most in need with resources that can be leveraged effectively towards health-promoting diet and physical activity.

## 2 Methods

### 2.1 Description of data and study design

The GLOBE (Dutch acronym for “Health and Living Conditions of the Population of Eindhoven and surroundings”) study is a prospective cohort study about understanding socioeconomic inequalities in health, conducted in Eindhoven and surrounding municipalities in the Netherlands (Van Lenthe et al., 2014). The 2014 GLOBE survey included multiple measures of economic, social, and embodied cultural capital, so data from this cross-sectional sample of participants aged between 25 and 75 years (N = 2812) were used in the analyses described in this paper.

### 2.2 Description of measures

#### 2.2.1 Forms of capital

Bourdieu considered social position to be a relational construct, in that people's social position depends on the amount of capital they possess relative to others rather than on the absolute amount of capital they possess (Pinxten & Lievens, 2014). Relative overall measures of each form of capital, operationalized as quartiles, were calculated to capture the forms of capital as relational constructs. The mean capital scores closest to the quartile cutoffs of the distributions were used as cutoff points for the quartile indicators.

##### 2.2.1.1 Economic capital

Three measures of economic capital reflecting material assets that can be converted into money or institutionalized as property rights were included in the analyses (Bourdieu, 1986): household equivalent income, financial strain, and housing tenure. Monthly household equivalent income was calculated by dividing reported monthly household income by the square root of the number of people living off this income. Financial strain was measured by two questions about having difficulties paying for essentials like food, rent, mortgage, and electricity in the past month (answer options were “no difficulty at all”, “some difficulty”, and “great difficulty”) and about how the respondent's household was able to make ends meet (answer options were “with great difficulty”, “with some difficulty”, “fairly easily”, and “easily”). Housing tenure was measured by asking respondents whether they own their home, rent through the private sector, or rent through the public sector.

To calculate a relative overall measure of economic capital, all measures were transformed to a scale ranging from 1 to 4 (e.g., for housing tenure, 1 = renting through public sector, 2.5 = renting through private sector, 4 = owning home). A mean economic capital score was calculated based on household equivalent income, an average of the two measures of financial strain, and housing tenure. This mean score was divided into quartiles to derive the relative overall measure of economic capital.

##### 2.2.1.2 Social capital

Social capital was measured by three health behavior-specific qualities of respondents' support network, using statements adapted from measures of descriptive and injunctive norms (Park & Smith, 2007). The associations of more general measures of social capital (e.g., size of network) with diet and physical activity were expected to be inconsistent and highly dependent on the behavior of, beliefs of, and stimulation from the people in one's social network. For this reason, we used measures of the activation of social capital towards a specific behavior (diet or physical activity). For each health behavior (diet or physical activity), respondents were asked to what extent they agreed with three statements about behavior of, beliefs of, and stimulation from their support network (e.g., “Most people who are important to you think you should eat healthily”). Answer choices were on a five-point scale ranging from “totally agree” to “totally disagree”.

For diet- and physical activity-related social capital, the mean of the three measures of social capital was calculated. Each of these two behavior-specific mean scores was divided into quartiles, resulting in relative overall measures of diet- and physical activity-related social capital.

##### 2.2.1.3 Embodied cultural capital

Cultural participation and reading frequency are two commonly used measures of embodied cultural capital as defined by Bourdieu (e.g. (Christensen & Carpiano, 2014; Pampel, 2011),). These measures are manifestations of cultural knowledge, values, and skills developed through socialization as high status cultural behaviors and signals used for cultural and social exclusion in a specific cultural setting (here, the Netherlands) (DiMaggio, 1982; Kraaykamp & van Eijck, 2010; Sullivan, 2001). Respondents reported how frequently they visited museums, music, dance, or theatre performances, and cultural monuments; answer options were “never”, “around once per year”, and “multiple times per year”. Respondents indicated how frequently they read books on a five-level scale ranging from “at least one book per week” to “never”.

The two measures of embodied cultural capital were averaged to calculate a mean overall embodied cultural capital score. This mean score was divided into quartiles to derive the relative overall measure of embodied cultural capital.

#### 2.2.2 Healthy diet and physical activity

Participation in sports and leisure time walking or cycling were included as healthy physical activity outcomes. Both types of physical activity were measured using the validated Short QUestionnaire to ASsess Health-enhancing physical activity (SQUASH) (Wendel-Vos et al., 2003). Respondents were asked to report the average frequency, duration, and intensity (light, moderate, or intense) of their weekly physical activity. For sports participation, respondents filled in up to four specific sports they practiced in recent months along with the frequency, duration, and intensity of their weekly participation in each sport. The respondents' self-reported intensities were combined with sport-specific intensity weights based on Ainsworth's categorization to derive the weekly time and intensity spent participating in sports (Ainsworth et al., 1993). The two types of physical activity were analyzed as separate dichotomous variables in order to identify potential differences in how capital influences each type of physical activity, with 1 = meaningfully engaging in physical activity and 0 = not meaningfully engaging in physical activity. In line with existing research (Oude Groeniger, Kamphuis, Mackenbach, Beenackers, & van Lenthe, 2019), participants meaningfully engaged in each type of physical activity if they reported spending at least 30 min once per week at moderate intensity on any given activity (e.g., leisure time cycling). This cutoff represents an amount of time that requires making an effort to engage in an activity.

Information on fruit and vegetable consumption was collected by asking (in two separate questions) how often, on average, participants consumed a serving of fruit or vegetables in the past month. Based on the Dutch Dietary Guidelines issued by the Health Council of the Netherlands, fruit and vegetable recommendations were met if a respondent reported eating at least two pieces of fruit every day and at least four servings of 50 g of vegetables every day, respectively (Gezondheidsraad, 2015). Fruit and vegetable consumption were analyzed as two separate dichotomous variables, with 1 = meeting recommended consumption levels and 0 = not meeting recommended consumption levels.

#### 2.2.3 Potential confounders

Age (in years), gender (male or female), country of birth (the Netherlands or other), and occupation type (full-time employment, part-time employment, retired, homemaker, unemployed, or other) were included in the analyses as potential confounders.

### 2.3 Statistical analysis

Multiple imputation was employed to address the potential bias introduced by missing data; twenty imputed datasets were generated based on an efficient predictor set (van Buuren, 2018). The analyses were performed on each imputed dataset, and results were pooled according to Rubin's rule. For each outcome, respondents with missing values for that outcome were excluded. Respondent-level cross-sectional survey weights were applied to the analyses to account for the GLOBE study sampling strategy.

Multiple logistic regression analyses were used to estimate the associations between the three forms of capital and meeting recommendations for healthy diet (fruit consumption, vegetable consumption) and meaningfully engaging in physical activity (sports participation, leisure time walking or cycling). A first set of models estimated the separate associations (referred to as the ‘main effects models’) of economic, social, and embodied cultural capital with each outcome (Models 1). Two-way interactions were added to Models 1: between economic and embodied cultural capital (Models 2A), between social and embodied cultural capital (Models 2B), and between economic and social capital (Models 2C). All models described in the main text were adjusted for age, gender, country of birth, and occupation type. Unadjusted models are presented in Supplementary File 1.

The forms of capital were operationalized as dummy variables representing each quartile, with the first (lowest) quartile as the reference category. Regression model coefficients are presented on the odds ratio scale. The added explanatory value of the models including two-way interactions was assessed using nested model analysis of variance (ANOVA) tests, which were run on each set of imputed models then averaged. Predicted probabilities were calculated to compare the main effects models and the models including two-way interactions between the forms of capital that statistically significantly added explanatory value (according to the nested model ANOVA tests). All analyses were performed in R (version 3.6.0).

## 3 Results

### 3.1 Descriptive statistics

Descriptive statistics from the non-imputed data for the full unweighted sample are shown in Table 1. The sample contained more females than males. The average age was 48.9 years. More than half of respondents meaningfully engaged in sports participation and leisure time walking or cycling, whereas a minority of respondents met recommendations for fruit consumption and vegetable consumption.

**Table 1:** Descriptive statistics of the unweighted GLOBE 2014 sample.

Variable	Total sample, N = 2812
Gender, %	
Woman	55.2
Man	44.8
Age (range: 25–75), mean (SD)	48.9 (15.6)
Birth country, %	
The Netherlands	88.2
Other	11.3
Missing values	0.5
Employment type, %	
Full-time employment	34.5
Part-time employment	25.7
Retired	22.1
Homemaker	4.7
Unemployed	7.9
Other	3.2
Missing values	1.9
Meaningfully engaging in sports participation, %	
Yes	56.8
No	35.3
Missing values	7.9
Meaningfully engaging in leisure time walking or cycling, %	
Yes	62.6
No	35.2
Missing values	2.2
Meeting fruit consumption recommendations, %	
Yes	33.0
No	61.9
Missing values	5.0
Meeting vegetable consumption recommendations, %	
Yes	21.6
No	74.3
Missing values	4.2
Economic capital, %	
Quartile 1 (lowest)	23.7
Quartile 2	25.4
Quartile 3	16.2
Quartile 4 (highest)	21.6
Missing values	13.1
Diet-related social capital, %	
Quartile 1 (lowest)	31.4
Quartile 2	16.9
Quartile 3	23.7
Quartile 4 (highest)	13.3
Missing values	14.7
Physical activity-related social capital, %	
Quartile 1 (lowest)	22.9
Quartile 2	29.3
Quartile 3	20.3
Quartile 4 (highest)	12.3
Missing values	15.2
Embodied cultural capital, %	
Quartile 1 (lowest)	26.1
Quartile 2	31.1
Quartile 3	28.1
Quartile 4 (highest)	12.5
Missing values	2.2

SD: standard deviation.

### 3.2 Healthy physical activity model results

#### 3.2.1 Sports participation

The main effects model estimates (Model 1, Table 2) show that all three forms of capital were positively associated with the likelihood of participating in sports. Nested model ANOVA tests indicated that the model including interactions between economic and social capital (Model 2C) explained more about the data than the main effects model (Model 1), while models with interactions between economic and embodied cultural capital (Model 2A) and social and embodied cultural capital (Model 2B) did not. Results from Model 1 and Model 2C are presented in Table 2, and full results (including from models 2A and 2B) are available in Supplementary File 2.

**Table 2:** Regression model results: sports participation.

Model	Main effects model (Model 1)			Economic capital x Social capital model (Model 2C)			
	Odds Ratio	95% CI		Odds Ratio	95% CI		Total Odds Ratio^a^
Variable		Lower	Upper		Lower	Upper	
ANOVA test (p-value)*				***0.04***			
Intercept	**1.82**	1.07	3.10	**2.59**	1.43	4.72	
*Economic capital*							
Quartile 1 (lowest)	1.00						
Quartile 2	**1.72**	1.32	2.23	1.02	0.62	1.67	
Quartile 3	**2.09**	1.53	2.86	1.44	0.84	2.47	
Quartile 4 (highest)	**2.36**	1.75	3.17	1.56	0.90	2.70	
*Social capital*							
Quartile 1 (lowest)	1.00						
Quartile 2	1.20	0.93	1.54	0.84	0.49	1.43	
Quartile 3	**2.08**	1.57	2.75	1.19	0.71	1.99	
Quartile 4 (highest)	**1.75**	1.28	2.40	0.97	0.53	1.78	
*Embodied cultural capital*							
Quartile 1 (lowest)	1.00						
Quartile 2	**1.79**	1.40	2.30	**1.77**	1.38	2.26	
Quartile 3	**2.09**	1.61	2.73	**2.08**	1.60	2.70	
Quartile 4 (highest)	**1.85**	1.32	2.58	**1.78**	1.27	2.48	
*Economic capital x Social capital*							
Quartile 2 x Quartile 2				1.93	0.96	3.89	
Quartile 2 x Quartile 3				**2.46**	1.18	5.12	2.98
Quartile 2 x Quartile 4				1.93	0.87	4.29	
Quartile 3 x Quartile 2				1.65	0.75	3.61	
Quartile 3 x Quartile 3				1.32	0.57	3.06	
Quartile 3 x Quartile 4				**3.07**	1.11	8.50	4.30
Quartile 4 x Quartile 2				1.33	0.63	2.81	
Quartile 4 x Quartile 3				**2.71**	1.22	6.05	5.04
Quartile 4 x Quartile 4				2.43	0.97	6.11	

For all three forms of capital, the reference category is quartile 1, the lowest quartile. All models were adjusted for age, gender, country of birth, and type of employment. Statistically significant estimates based on 95% confidence intervals are indicated in **bold**.

*The nested model ANOVA tests compared each model containing interaction terms with the main effects model. A model containing interaction terms can be said to explain more about the data than the main effects model if the ANOVA test p-value <0.05. ANOVA test p-values <0.05 are indicated in bold.

ANOVA: analysis of variance; CI: confidence interval.

a Interpreting the influence of the interaction effects on the likelihood of participating in sports requires calculating what we call the ‘total odds ratio’. For a given combination of economic and social capital, the total odds ratio refers to the exponent of the sum of (1) the log odds for having a given level of economic capital and a level of social capital in the first quartile, (2) the log odds for having a given level of social capital and a level of economic capital in the first quartile, and (3) the log odds for having given levels of both economic and social capital. Recall that the log odds = ln(odds ratio) and odds ratio = exp(log odds). For example, the total odds ratio of having a level of economic capital in the fourth quartile and a level of social capital in the third quartile is $e^{\ln\left(1.56\right)+\ln\left(1.19\right)+\ln\left(2.71\right)}=e^{0.45+0.18+1.00}=e^{1.62}=5.04$.

Several estimated interactions between quartiles of economic and social capital were statistically significant (Model 2C in Table 2). Odds ratios of these statistically significant interactions are larger than 1, showing that having specific higher levels of both economic and social capital boosted the likelihood of participating in sports relative to having higher levels of just one or the other.

**Fig. 1:**
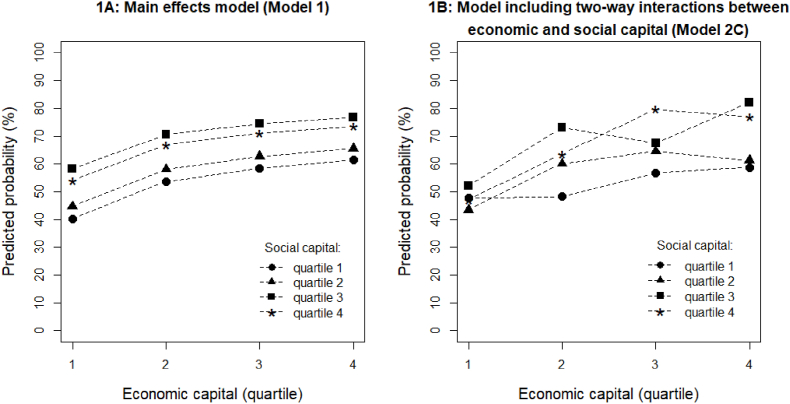
Title: Predicted probabilities of participating in sports.

Predicted probabilities of participating in sports for all combinations of economic and social capital are presented in Fig. 1. All other variables were held constant at gender = female, age = 50 years, birth country = the Netherlands, employment type = full-time, cultural capital = quartile 4 (highest). Fig. 1A illustrates that in the main effects model, the association between economic capital and the probability of participating in sports is the same for all levels of social capital; the curves for each level of social capital are parallel. When accounting for two-way interactions between economic and social capital (Fig. 1B), the association between social capital and the probability of participating in sports depends on the level of economic capital; this is visualized by the nonparallel social capital curves. If economic and social capital were not conditional on each other, we would expect to see parallel curves even when including the two-way interactions between economic and social capital in the model. The nonparallel curves in Fig. 1B illustrate how the association of economic capital with sports participation changes depending on the relative level of social capital; for example, the slopes between having a relative level of economic capital in quartiles 2 and 3 are increasing for relative levels of social capital in quartiles 1, 2, and 4 but are decreasing for a relative level of social capital in quartile 3 (Fig. 1B), whereas these slopes are identical for all relative levels of social capital in Fig. 1A. Comparing Fig. 1A and B, we can see how having certain combinations of economic and social capital boosts their associations with sports participation. For example, someone with economic capital in quartile 4 and social capital in quartile 3 (holding all other variables constant as described above) has about a 75% probability of participating in sports when these forms of capital are assumed to be associated with sports participation independent of each other (Fig. 1A) but about an 80% chance according to the model including the two-way interactions (Fig. 1B). The model including the interaction effects more accurately reflects the relationships, as evidenced by the ANOVA test result (see Table 2) and the nonparallel curves in Fig. 1B.

Fig. 1 caption: Fig. 1 shows the predicted probabilities of participating in sports for the main effects model (Fig. 1A) and the model including two-way interactions between economic and social capital (Fig. 1B).

#### 3.2.2 Leisure time walking or cycling

The main effects model estimates (Table 3) show that higher levels of embodied cultural capital were associated with an increased likelihood of walking or cycling in leisure time. Economic and social capital were not associated with the likelihood of walking or cycling in leisure time.

**Table 3:** Regression model results: leisure time walking or cycling.

Model	Main effects model (Model 1)		
	Odds Ratio	95% CI	
Variable		Lower	Upper
Intercept	**0.13**	0.08	0.22
*Economic capital*			
Quartile 1 (lowest)	1.00		
Quartile 2	0.91	0.71	1.18
Quartile 3	0.92	0.67	1.26
Quartile 4 (highest)	1.17	0.86	1.57
*Physical activity-related social capital*			
Quartile 1 (lowest)	1.00		
Quartile 2	1.15	0.90	1.46
Quartile 3	1.27	0.98	1.65
Quartile 4 (highest)	0.97	0.71	1.32
*Embodied cultural capital*			
Quartile 1 (lowest)	1.00		
Quartile 2	**1.57**	1.24	2.00
Quartile 3	**2.03**	1.56	2.62
Quartile 4 (highest)	**3.42**	2.37	4.96

For all three forms of capital, the reference category is quartile 1, the lowest quartile. All models were adjusted for age, gender, country of birth, and type of employment. Statistically significant estimates based on 95% confidence intervals are indicated in **bold**.

CI: confidence interval.

For leisure time walking or cycling, nested model ANOVA tests indicated that models containing two-way interactions between the forms of capital (Models 2A, 2B, and 2C) did not explain more about the data than the main effects model (Model 1); full results for Models 2A, 2B, and 2C are presented in Supplementary File 2.

### 3.3 Healthy fruit and vegetable consumption model results

In the main effects models (Table 4), all three forms of capital were positively associated with the likelihood of meeting vegetable consumption recommendations, and embodied cultural capital and social capital were positively associated with the likelihood of meeting fruit consumption recommendations. For both fruit and vegetable consumption, embodied cultural capital had stronger associations with the likelihood of meeting recommendations than social and economic capital.

**Table 4:** Regression model results: healthy fruit and vegetable consumption.

Outcome	Fruit consumption: Main effects model (Model 1)			Vegetable consumption: Main effects model (Model 1)		
	Odds Ratio	95% CI		Odds Ratio	95% CI	
Variable		Lower	Upper		Lower	Upper
Intercept	**0.09**	0.05	0.16	**0.17**	0.10	0.31
*Economic capital*						
Quartile 1 (lowest)	1.00					
Quartile 2	1.03	0.79	1.35	1.36	0.99	1.86
Quartile 3	0.97	0.71	1.32	**1.49**	1.04	2.12
Quartile 4 (highest)	1.24	0.93	1.66	**1.41**	1.00	1.98
*Diet-related social capital*						
Quartile 1 (lowest)	1.00					
Quartile 2	1.29	0.98	1.70	1.33	0.99	1.80
Quartile 3	**1.57**	1.23	2.01	1.30	0.99	1.72
Quartile 4 (highest)	**1.82**	1.37	2.43	**1.54**	1.13	2.10
*Embodied cultural capital*						
Quartile 1 (lowest)	1.00					
Quartile 2	**1.43**	1.10	1.86	**1.56**	1.15	2.13
Quartile 3	**1.67**	1.28	2.18	**2.22**	1.64	3.01
Quartile 4 (highest)	**2.61**	1.90	3.58	**3.01**	2.11	4.30

For all three forms of capital, the reference category is quartile 1, the lowest quartile. All models were adjusted for age, gender, country of birth, and type of employment. Statistically significant estimates based on 95% confidence intervals are indicated in bold.

CI: confidence interval.

Nested model ANOVA tests showed that models containing two-way interactions between the forms of capital (Models 2A, 2B, and 2C) did not explain more about the data than the main effects models (Models 1) for either fruit or vegetable consumption (see Supplementary File 3 for results for Models 2A, 2B, and 2C for each outcome).

## 4 Discussion

### 4.1 Summary of findings

In our investigation of separate associations of the three forms of capital with diet and physical activity, economic, social, and embodied cultural capital were positively associated with the likelihood of participating in sports and meeting vegetable consumption recommendations. Social and embodied cultural capital were positively associated with meeting fruit consumption recommendations, and only embodied cultural capital was positively associated with leisure time walking or cycling.

Regarding this study's primary hypotheses about interactions between the forms of capital, economic and social capital were conditional on each other in their association with the likelihood of participating in sports; having specific higher levels of both economic *and* social capital boosted their influence on sports participation compared to having higher levels of just one *or* the other. Contrary to our hypotheses, no other two-way combinations of the forms of capital were conditional on each other for any of the outcomes investigated in this study.

### 4.2 Interpretation of findings

Relative to economic and social capital, embodied cultural capital had a more consistent association with all health behavior outcomes in this study, showing that embodied cultural capital is an important mechanism for both diet and physical activity. Cultural participation is a well-known indicator of social distinction, and health behavior may also be a matter of taste and a tool for social distinction (Oude Groeniger et al., 2017, Oude Groeniger, Kamphuis, Mackenbach, Beenackers, & van Lenthe, 2019). This finding is in line with several recent studies that investigated cultural capital and health behavior (Hashemi et al., 2018 Kamphuis, Oude Groeniger, & van Lenthe, 2018; Mollborn & Modile, 2022; Oncini & Guetto, 2018).

The prominence of free, convenient walking and cycling infrastructure along with other elements of walkability and cyclability, such as high population density and land-use mix, in the Netherlands (Lam et al., 2022) may explain why economic and social capital were not associated with leisure time walking or cycling. This type of physical activity is free and is built (both physically and in terms of social norms) into the Dutch societal structure. For this reason, these findings may not generalize to contexts beyond the Netherlands. One surprising finding from this study is that the associations of the three forms of capital differed between fruit and vegetable consumption, as economic capital was associated with vegetable consumption but not with fruit consumption. Many studies operationalize fruit and vegetable consumption as a single outcome (e.g. (Basu et al., 2021; Lallukka et al., 2010),), but our findings show that these two types of dietary intake may be driven by somewhat distinct mechanisms. Fruit generally contains more sugar and is easier to prepare than vegetables, which may make eating fruit feel like more of a treat than eating vegetables (Breslin, 2013; Pollard et al., 2002), regardless of the economic resources one has access to. This finding aligns with a study conducted in the US that observed greater income disparities in vegetable consumption than in fruit consumption (Grimm et al., 2012).

We found some evidence that economic and social capital are conditional on each other in their association with sports participation. More specifically, we found that having specific higher levels of economic capital combined with specific higher levels of social capital boosted the likelihood of participating in sports. Unlike other forms of physical activity, participating in sports often involves an economic cost (e.g., fitness club membership, fee to join a sports team), making economic capital a barrier or facilitator to sports participation. Sports participation may also be a more discretionary activity than food intake or leisure time walking or cycling, so social support combined with economic means may be especially useful in encouraging one to spend their finite time on sports participation.

Contrary to our expectations, the association of embodied cultural capital with diet and physical activity was not conditional on levels of economic or social capital. This finding adds nuance to existing literature on the relationship between cultural capital and health behavior and suggests that embodied cultural capital appears to be a mechanism operating separately from the labor market (Savage et al., 2015) or other types of economic and social capital. Indeed, cultural values have been acknowledged as a source of power that, in the context of diet and physical activity, can generate empowerment to behave in more health-promoting ways (McCartney et al., 2021). While the development of embodied cultural capital may also take place later in adulthood (Aschaffenburg & Maas, 1997), it has primarily been theorized as a long-lasting process starting early in life that is shaped by factors such as parental education, childrearing practices, navigating institutions of higher education, and engagement with highbrow cultural goods and activities (e.g., art and books) (Kraaykamp & van Eijck, 2010). As a long-lasting process, the association of embodied cultural capital with health behavior may be independent of factors such as income in adulthood and social support.

### 4.3 Methodological considerations

Several limitations of this study's methodological approach should be kept in mind when interpreting the results. The use of cross-sectional data leaves open the possibility of reverse causality. Capital and health behavior may interact in a vicious cycle for those with fewer resources and in a virtuous cycle for those with more resources, as was postulated by Weaver et al. (Weaver et al., 2014) in the context of diabetes self-management. This may apply especially to behavior-specific social capital; for example, those who spend more time on sports may be more likely to develop a network consisting of people who also play sports. We also cannot rule out that some of the observed relationships are actually due to capital accumulation throughout the life course (Bourdieu, 1986), which we were not able to test with cross-sectional data. While our findings are a useful starting point for considering how the forms of capital are (and are not) interrelated in their associations with diet and physical activity, it is imperative to complement this research with studies that include feedback loops between forms of capital and health behavior over the life course, using longitudinal data and complex systems methods, to consider the conversion, accumulation, and transmission of capital.

The outcome variables were dichotomized for two main reasons: the physical activity variables were heavily skewed towards zero (most respondents who did not meaningfully engage in sports or leisure time walking or cycling recommendations reported not engaging in that type of physical activity at all) and the relationship between the behaviors and health may not be linear (consuming more fruits or vegetables is only healthy up to a certain point, after which it becomes unhealthy). However, we acknowledge that dichotomizing the variables may have obscured some detail in the observed relationships.

In constructing the overall relative measures of capital, we aimed to strike a balance between adhering to Bourdieu's conceptualizations of the forms of capital and using available, established measures that could help answer our research questions. This approach was helpful for providing insight into how the forms of capital are conditional on each other in the context of diet and physical activity in the Netherlands based on Bourdieu's theory. However, this approach neglects the complexity of the conceptualization and measurement of the forms of capital, especially social and embodied cultural capital. Though the data was not available, a more complete measure of social capital for this study could have considered the *size* of the participants' health behavior-specific support network in addition to behavior-specific qualities of the network, as size of network is another key component of social capital as defined by Bourdieu. The measures of embodied cultural capital included in this study, reading frequency and cultural participation, are well-established measures of embodied cultural capital that align closely with Bourdieu's conceptualization. It is important to acknowledge that this conceptualization, which has been criticized for its narrow view of highbrow cultural capital as a tool for social distinction, was introduced and is largely maintained by people who possess higher levels of capital according to these definitions (Bourdieu, 1984; Peterson & Kern, 1996). Applying Bourdieu's conceptualization of embodied cultural capital reduces it to a static and universally applicable concept, while in reality, embodied cultural capital is likely evolving and context-specific (Dijkstra & Horstman, 2023). Especially since the results of this study suggest that embodied cultural capital has a strong, consistent association with health behavior, future research should consider the construction of the social norms that dictate what knowledge, skills, and values serve as embodied cultural capital and how that construction of norms may impact the relationships between the forms of capital and health behaviors (Dijkstra & Horstman, 2023). In doing so, a definition of cultural capital that transcends highbrow cultural capital to encompass a larger diversity of forms of cultural expression and account for specific cultural contexts could reflect the complexity of embodied cultural capital in a more realistic and inclusive way.

A final methodological consideration is that our study focused on how structural resources are associated with health behavior but did not explicitly consider agency or other individual-level determinants of behavior. Bourdieu's capital theory focuses on understanding why and how social position tends to persist, but the individual's capacity for behavior change in the context of their environment is crucial to study if we want to foster environments that are conducive to healthier behavior (Cockerham, 2005). Empirical studies that explicitly account for both agency and the resources one has access to would complement the growing body of theoretical literature on this topic (Abel & Frohlich, 2012; Cockerham, 2005; Frohlich & Potvin, 2010; Rütten & Gelius, 2011). Our findings exhibited the general pattern that having higher levels of capital is associated with healthier diet and physical activity behavior, though there was some nuance in the magnitudes of the relationships between certain levels of capital and the outcomes that we did not analyze. It is possible that other individual-level behavior change mechanisms, such as susceptibility to social norms, may be driving these relationships, which may warrant further research supported by behavior change theories.

### 4.4 Policy implications

The strong and consistent association of embodied cultural capital with all outcomes investigated in this study signals that policy addressing embodied cultural capital is warranted. Given that embodied cultural capital may be largely developed early in life, programs in the school setting, such as the provision of healthy school lunches (Oosterhoff et al., 2020), may facilitate the development of knowledge, values, and skills about healthy behavior that last throughout adulthood. Because embodied cultural capital results from power dynamics within societies and is gradually cultivated and internalized over time through socialization processes, efforts to intervene on it in adulthood may be elusive (McCartney et al., 2021). That said, embodied cultural capital should be thoughtfully considered in policies aimed at increasing healthy behavior. A first approach to this is for policymakers to develop interventions alongside members of the groups targeted by the interventions, so that the implications of differences in levels of embodied cultural capital are incorporated into the policy design. In this way, policies will be better aligned with what is meaningful and feasible for those in the target group (rather than the policymakers’ perception of what is meaningful and feasible for those in the target group) and may circumvent undesired effects of policies such as feelings of paternalization, stigmatization, and feeling looked down upon, which could lead to low policy effectiveness and other unintended consequences (van Meurs, Oude Groeniger, de Koster, & van der Waal, 2022). A second approach to accounting for embodied cultural capital in public health policy is to make structural, environmental changes that dampen the importance of embodied cultural resources for diet and physical activity, especially when what constitutes embodied cultural resources for policymakers does not align with the social norms and cultural context of the target population. For example, the food environment could be altered by increasing the availability and promotion of healthy foods (i.e., fruits and vegetables) and reducing the availability and promotion of unhealthy foods (i.e., highly-processed foods), to weaken the dependence between healthy food choices and embodied cultural capital. This type of approach has been successful in other domains, such as smoking bans contributing to decreasing smoking rates in Europe (Spinney, 2007).

Based on our finding that economic and social capital are conditional on each other in their influence on sports participation, interventions providing both social and economic support may be especially effective for increasing this health behavior. The provision of collective housing, which is characterized by more communal spaces and collectively organized facilities than conventional housing (Vestbro, 2000), is one example of this type of intervention. The housing itself can be considered economic capital, and shared facilities (e.g., laundry) often make collective housing more economical. Collective housing complexes that use their outdoor communal spaces to organize social events could generate physical activity-specific social capital, which could make such an intervention especially conducive to participation in sports.

### 4.5 Conclusions

This study was the first to investigate how economic, social, and embodied cultural capital are conditional on each other in their associations with diet and physical activity for adults in the Netherlands. We found that having a combination of higher economic and social resources boosted sports participation compared to having higher levels of only one of these forms of capital; other combinations of capital were not found to be conditional on each other. Embodied cultural capital was consistently positively associated with all diet and physical activity outcomes, though these associations were not conditional on economic or social capital. Economic and social capital were positively associated with some, but not all, outcomes. Interventions that offer both economic and social support may be especially effective for increasing sports participation, and policies that account for differences in embodied cultural capital or changes to the environment that dampen the importance of embodied cultural resources for healthy behavior may help improve both diet and physical activity.

## Ethical statement

This study was conducted according to the guidelines laid down in the Declaration of Helsinki. The use of personal data in the GLOBE study is in compliance with the Dutch Personal Data Protection Act, and the Municipal Database Act and has been registered with the Dutch Data Protection Authority (number 1248943). In accordance with the Dutch law for medical-scientific research, no formal approval was required for this non-invasive survey research.

## Credit author statement

**Andrea L. Mudd**: Conceptualization, Methodology, Formal analysis, Writing – Original Draft, Writing – Review & Editing. **Joost Oude Groeniger**: Conceptualization, Methodology, Data Curation, Writing – Review & Editing. **Michèlle Bal**: Conceptualization, Methodology, Writing – Review & Editing.

**Sanne E. Verra**: Writing – Review & Editing. **Frank J. van Lenthe**: Conceptualization, Data Curation, Writing – Review & Editing. **Carlijn B. M. Kamphuis**: Conceptualization, Methodology, Writing – Review & Editing, Supervision, Funding acquisition.

## Financial disclosure statement

Andrea L. Mudd, Sanne E. Verra, and Carlijn B.M. Kamphuis were supported by the Innovational Research Incentives Scheme (Vl.Vidi.198.001), financed by the 10.13039/501100003246Netherlands Organization for Scientific Research (NWO). The funding body had no role in the design of the study; collection, analysis, and interpretation of data; or in writing the manuscript.

## Declaration of competing interest

Declaration of interest: none.

## Data Availability

Data will be made available on request.

## Appendix A Supplementary data

Supplementary File 1 Regression model results for unadjusted models Supplementary File 1

Supplementary File 2 Full regression model results for sports participation and leisure time walking or cycling Supplementary File 2

Supplementary File 3 Full regression model results for fruit consumption and vegetable consumption Supplementary File 3
