# Supplementary material for: Testing conditionality with Bourdieu's capital theory: How economic, social, and embodied cultural capital are associated with diet and physical activity in the Netherlands
Source: SSM Popul Health. 2023 Apr 10;22:101401. doi: 10.1016/j.ssmph.2023.101401 (PMC10139966; doi:10.1016/j.ssmph.2023.101401)
Supplement: Supplementary File 1 — Regression model results for unadjusted models [file mmc1.docx]

# Supplementary File 1: Unadjusted model results

*Healthy physical activity*

*Sports participation*

Table 1. Unadjusted regression model results: sports participation

| Model | Main effects model  (Model 1) | | | Economic capital x Embodied cultural capital model  (Model 2A) | | | Social capital x Embodied cultural capital model  (Model 2B) | | | Economic capital x Social capital model  (Model 2C) | | |
| --- | --- | --- | --- | --- | --- | --- | --- | --- | --- | --- | --- | --- |
|  | Odds Ratio | 95% CI | | Odds Ratio | 95% CI | | Odds Ratio | 95% CI | | Odds Ratio | 95% CI | |
| Variable |  | Lower | Upper |  | Lower | Upper |  | Lower | Upper |  | Lower | Upper |
| *ANOVA test (p-value)^*^* |  |  |  | *0.252* |  |  | *0.238* |  |  | ***0.036*** |  |  |
| Intercept | **0.44** | 0.34 | 0.58 | **0.40** | 0.29 | 0.55 | **0.44** | 0.31 | 0.63 | **0.63** | 0.44 | 0.89 |
| *Economic capital* | |  |  |  |  |  |  |  |  |  |  |  |
| Quartile 1 (lowest) | 1.00 |  |  |  |  |  |  |  |  |  |  |  |
| Quartile 2 | **1.94** | 1.52 | 2.48 | **2.34** | 1.54 | 3.55 | **1.95** | 1.53 | 2.49 | 1.17 | 0.74 | 1.87 |
| Quartile 3 | **2.43** | 1.81 | 3.25 | **3.26** | 1.83 | 5.78 | **2.45** | 1.83 | 3.29 | 1.62 | 0.98 | 2.67 |
| Quartile 4 (highest) | **2.64** | 2.01 | 3.47 | **2.36** | 1.33 | 4.19 | **2.65** | 2.01 | 3.48 | **1.69** | 1.01 | 2.84 |
| *Social capital* | |  |  |  |  |  |  |  |  |  |  |  |
| Quartile 1 (lowest) | 1.00 |  |  |  |  |  |  |  |  |  |  |  |
| Quartile 2 | 1.15 | 0.90 | 1.47 | 1.18 | 0.92 | 1.51 | 1.35 | 0.85 | 2.14 | 0.83 | 0.50 | 1.36 |
| Quartile 3 | **1.90** | 1.45 | 2.47 | **1.93** | 1.48 | 2.52 | **1.65** | 1.02 | 2.67 | 1.06 | 0.65 | 1.74 |
| Quartile 4 (highest) | **1.58** | 1.17 | 2.13 | **1.63** | 1.21 | 2.21 | 1.36 | 0.77 | 2.40 | 0.86 | 0.49 | 1.50 |
| *Embodied cultural capital* | |  |  |  |  |  |  |  |  |  |  |  |
| Quartile 1 (lowest) | 1.00 |  |  |  |  |  |  |  |  |  |  |  |
| Quartile 2 | **1.82** | 1.43 | 2.31 | **2.10** | 1.38 | 3.20 | **1.95** | 1.25 | 3.04 | **1.79** | 1.41 | 2.27 |
| Quartile 3 | **2.02** | 1.57 | 2.60 | **2.07** | 1.26 | 3.40 | **1.72** | 1.08 | 2.74 | **2.01** | 1.56 | 2.59 |
| Quartile 4 (highest) | **1.45** | 1.06 | 1.99 | **2.42** | 1.16 | 5.08 | 1.65 | 0.84 | 3.26 | **1.39** | 1.01 | 1.91 |
| *Economic capital x Embodied cultural capital* | | | |  |  |  |  |  |  |  |  |  |
| Quartile 2 x Quartile 2 | | |  | 0.67 | 0.37 | 1.20 |  |  |  |  |  |  |
| Quartile 2 x Quartile 3 | | |  | 0.91 | 0.47 | 1.77 |  |  |  |  |  |  |
| Quartile 2 x Quartile 4 | | |  | 0.66 | 0.25 | 1.73 |  |  |  |  |  |  |
| Quartile 3 x Quartile 2 | | |  | 0.84 | 0.39 | 1.81 |  |  |  |  |  |  |
| Quartile 3 x Quartile 3 | | |  | 0.81 | 0.36 | 1.80 |  |  |  |  |  |  |
| Quartile 3 x Quartile 4 | | |  | **0.29** | 0.10 | 0.83 |  |  |  |  |  |  |
| Quartile 4 x Quartile 2 | | |  | 1.14 | 0.53 | 2.44 |  |  |  |  |  |  |
| Quartile 4 x Quartile 3 | | |  | 1.20 | 0.55 | 2.61 |  |  |  |  |  |  |
| Quartile 4 x Quartile 4 | | |  | 0.77 | 0.29 | 2.04 |  |  |  |  |  |  |
| *Social capital x Embodied cultural capital* | | |  |  |  |  |  |  |  |  |  |  |
| Quartile 2 x Quartile 2 | | |  |  |  |  | 0.76 | 0.40 | 1.44 |  |  |  |
| Quartile 2 x Quartile 3 | | |  |  |  |  | 0.95 | 0.50 | 1.80 |  |  |  |
| Quartile 2 x Quartile 4 | | |  |  |  |  | 0.69 | 0.29 | 1.64 |  |  |  |
| Quartile 3 x Quartile 2 | | |  |  |  |  | 1.25 | 0.61 | 2.57 |  |  |  |
| Quartile 3 x Quartile 3 | | |  |  |  |  | 1.28 | 0.63 | 2.59 |  |  |  |
| Quartile 3 x Quartile 4 | | |  |  |  |  | 1.04 | 0.41 | 2.67 |  |  |  |
| Quartile 4 x Quartile 2 | | |  |  |  |  | 0.81 | 0.37 | 1.77 |  |  |  |
| Quartile 4 x Quartile 3 | | |  |  |  |  | **2.44** | 1.05 | 5.66 |  |  |  |
| Quartile 4 x Quartile 4 | | |  |  |  |  | 0.97 | 0.34 | 2.74 |  |  |  |
| *Economic capital x Social capital* | | | |  |  |  |  |  |  |  |  |  |
| Quartile 2 x Quartile 2 | | |  |  |  |  |  |  |  | 1.83 | 0.94 | 3.56 |
| Quartile 2 x Quartile 3 | | |  |  |  |  |  |  |  | 1.61 | 0.76 | 3.41 |
| Quartile 2 x Quartile 4 | | |  |  |  |  |  |  |  | 1.34 | 0.66 | 2.73 |
| Quartile 3 x Quartile 2 | | |  |  |  |  |  |  |  | **2.41** | 1.19 | 4.90 |
| Quartile 3 x Quartile 3 | | |  |  |  |  |  |  |  | 1.50 | 0.68 | 3.34 |
| Quartile 3 x Quartile 4 | | |  |  |  |  |  |  |  | **2.83** | 1.31 | 6.14 |
| Quartile 4 x Quartile 2 | | |  |  |  |  |  |  |  | 1.93 | 0.89 | 4.17 |
| Quartile 4 x Quartile 3 | | |  |  |  |  |  |  |  | **3.27** | 1.25 | 8.57 |
| Quartile 4 x Quartile 4 | | |  |  |  |  |  |  |  | **2.65** | 1.10 | 6.38 |

For all three forms of capital, the reference category is quartile 1, the lowest quartile. Statistically significant estimates based on 95% confidence intervals are indicated in **bold**.

^*^The nested model ANOVA tests compared each model containing interaction terms with the main effects model. A model containing interaction terms can be said to explain more about the data than the main effects model if the ANOVA test p-value < 0.05. ANOVA test p-values < 0.05 are indicated in **bold**.

ANOVA: analysis of variance; CI: confidence interval.

*Leisure time walking or cycling*

Table 2. Unadjusted regression model results: leisure time walking or cycling

| Model | Main effects model  (Model 1) | | | Economic capital x Embodied cultural capital model  (Model 2A) | | | Social capital x Embodied cultural capital model  (Model 2B) | | | Economic capital x Social capital model  (Model 2C) | | |
| --- | --- | --- | --- | --- | --- | --- | --- | --- | --- | --- | --- | --- |
|  | Odds Ratio | 95% CI | | Odds Ratio | 95% CI | | Odds Ratio | 95% CI | | Odds Ratio | 95% CI | |
| Variable |  | Lower | Upper |  | Lower | Upper |  | Lower | Upper |  | Lower | Upper |
| *ANOVA test (p-value)^*^* |  |  |  | *0.271* |  |  | *0.267* |  |  | *0.838* |  |  |
| Intercept | **1.29** | 1.01 | 1.65 | **1.39** | 1.04 | 1.86 | 1.30 | 0.95 | 1.78 | **1.48** | 1.06 | 2.07 |
| *Economic capital* | |  |  |  |  |  |  |  |  |  |  |  |
| Quartile 1 (lowest) | 1.00 |  |  |  |  |  |  |  |  |  |  |  |
| Quartile 2 | **0.70** | 0.55 | 0.89 | 0.74 | 0.50 | 1.09 | **0.69** | 0.54 | 0.88 | **0.58** | 0.37 | 0.90 |
| Quartile 3 | **0.70** | 0.53 | 0.93 | **0.48** | 0.28 | 0.82 | **0.69** | 0.52 | 0.91 | **0.60** | 0.37 | 0.97 |
| Quartile 4 (highest) | 0.90 | 0.68 | 1.17 | 0.65 | 0.38 | 1.13 | 0.89 | 0.68 | 1.17 | 0.76 | 0.45 | 1.26 |
| *Social capital* | |  |  |  |  |  |  |  |  |  |  |  |
| Quartile 1 (lowest) | 1.00 |  |  |  |  |  |  |  |  |  |  |  |
| Quartile 2 | 1.17 | 0.93 | 1.47 | 1.17 | 0.93 | 1.48 | 1.12 | 0.73 | 1.72 | 0.89 | 0.55 | 1.44 |
| Quartile 3 | **1.29** | 1.00 | 1.65 | **1.28** | 1.00 | 1.65 | **1.78** | 1.13 | 2.82 | 1.28 | 0.76 | 2.16 |
| Quartile 4 (highest) | 1.09 | 0.81 | 1.45 | 1.08 | 0.81 | 1.45 | 0.72 | 0.42 | 1.24 | 0.79 | 0.45 | 1.38 |
| *Embodied cultural capital* | |  |  |  |  |  |  |  |  |  |  |  |
| Quartile 1 (lowest) | 1.00 |  |  |  |  |  |  |  |  |  |  |  |
| Quartile 2 | **1.37** | 1.09 | 1.72 | 1.38 | 0.92 | 2.09 | 1.40 | 0.94 | 2.10 | **1.37** | 1.09 | 1.71 |
| Quartile 3 | **1.80** | 1.42 | 2.30 | 1.32 | 0.80 | 2.18 | **1.88** | 1.22 | 2.91 | **1.80** | 1.41 | 2.30 |
| Quartile 4 (highest) | **3.93** | 2.76 | 5.59 | **2.43** | 1.17 | 5.06 | **3.22** | 1.62 | 6.40 | **3.90** | 2.74 | 5.55 |
| *Economic capital x Embodied cultural capital* | | | |  |  |  |  |  |  |  |  |  |
| Quartile 2 x Quartile 2 | | |  | 0.72 | 0.41 | 1.28 |  |  |  |  |  |  |
| Quartile 2 x Quartile 3 | | |  | 1.31 | 0.68 | 2.52 |  |  |  |  |  |  |
| Quartile 2 x Quartile 4 | | |  | 1.58 | 0.58 | 4.29 |  |  |  |  |  |  |
| Quartile 3 x Quartile 2 | | |  | 1.66 | 0.79 | 3.46 |  |  |  |  |  |  |
| Quartile 3 x Quartile 3 | | |  | 1.83 | 0.85 | 3.91 |  |  |  |  |  |  |
| Quartile 3 x Quartile 4 | | |  | 2.47 | 0.81 | 7.52 |  |  |  |  |  |  |
| Quartile 4 x Quartile 2 | | |  | 1.36 | 0.66 | 2.80 |  |  |  |  |  |  |
| Quartile 4 x Quartile 3 | | |  | 1.80 | 0.84 | 3.86 |  |  |  |  |  |  |
| Quartile 4 x Quartile 4 | | |  | 2.05 | 0.74 | 5.64 |  |  |  |  |  |  |
| *Social capital x Embodied cultural capital* | | |  |  |  |  |  |  |  |  |  |  |
| Quartile 2 x Quartile 2 | | |  |  |  |  | 1.07 | 0.60 | 1.91 |  |  |  |
| Quartile 2 x Quartile 3 | | |  |  |  |  | 0.98 | 0.53 | 1.81 |  |  |  |
| Quartile 2 x Quartile 4 | | |  |  |  |  | 1.27 | 0.51 | 3.16 |  |  |  |
| Quartile 3 x Quartile 2 | | |  |  |  |  | 0.56 | 0.29 | 1.06 |  |  |  |
| Quartile 3 x Quartile 3 | | |  |  |  |  | 0.66 | 0.34 | 1.29 |  |  |  |
| Quartile 3 x Quartile 4 | | |  |  |  |  | 0.96 | 0.35 | 2.64 |  |  |  |
| Quartile 4 x Quartile 2 | | |  |  |  |  | 1.86 | 0.88 | 3.94 |  |  |  |
| Quartile 4 x Quartile 3 | | |  |  |  |  | 1.51 | 0.71 | 3.20 |  |  |  |
| Quartile 4 x Quartile 4 | | |  |  |  |  | 2.16 | 0.68 | 6.80 |  |  |  |
| *Economic capital x Social capital* | | | |  |  |  |  |  |  |  |  |  |
| Quartile 2 x Quartile 2 | | |  |  |  |  |  |  |  | 1.43 | 0.75 | 2.72 |
| Quartile 2 x Quartile 3 | | |  |  |  |  |  |  |  | 1.44 | 0.72 | 2.89 |
| Quartile 2 x Quartile 4 | | |  |  |  |  |  |  |  | 1.39 | 0.68 | 2.82 |
| Quartile 3 x Quartile 2 | | |  |  |  |  |  |  |  | 1.09 | 0.53 | 2.25 |
| Quartile 3 x Quartile 3 | | |  |  |  |  |  |  |  | 0.93 | 0.43 | 2.00 |
| Quartile 3 x Quartile 4 | | |  |  |  |  |  |  |  | 0.97 | 0.46 | 2.05 |
| Quartile 4 x Quartile 2 | | |  |  |  |  |  |  |  | 1.53 | 0.71 | 3.26 |
| Quartile 4 x Quartile 3 | | |  |  |  |  |  |  |  | 1.53 | 0.66 | 3.55 |
| Quartile 4 x Quartile 4 | | |  |  |  |  |  |  |  | 1.59 | 0.67 | 3.77 |

For all three forms of capital, the reference category is quartile 1, the lowest quartile. Statistically significant estimates based on 95% confidence intervals are indicated in **bold**.

^*^The nested model ANOVA tests compared each model containing interaction terms with the main effects model. A model containing interaction terms can be said to explain more about the data than the main effects model if the ANOVA test p-value < 0.05. ANOVA test p-values < 0.05 are indicated in **bold**.

ANOVA: analysis of variance; CI: confidence interval.

*Healthy diet*

*Fruit consumption*

Table 3. Unadjusted regression model results: fruit consumption

| Model | Main effects model  (Model 1) | | | Economic capital x Embodied cultural capital model  (Model 2A) | | | Social capital x Embodied cultural capital model  (Model 2B) | | | Economic capital x Social capital model  (Model 2C) | | |
| --- | --- | --- | --- | --- | --- | --- | --- | --- | --- | --- | --- | --- |
|  | Odds Ratios | 95% CI | | Odds Ratios | 95% CI | | Odds Ratios | 95% CI | | Odds Ratios | 95% CI | |
| Variable |  | Lower | Upper |  | Lower | Upper |  | Lower | Upper |  | Lower | Upper |
| *ANOVA test (p-value)^*^* |  |  |  | *0.357* |  |  | *0.858* |  |  | *0.278* |  |  |
| Intercept | **0.25** | 0.19 | 0.33 | **0.28** | 0.20 | 0.39 | **0.24** | 0.17 | 0.34 | **0.23** | 0.16 | 0.33 |
| *Economic capital* | |  |  |  |  |  |  |  |  |  |  |  |
| Quartile 1 (lowest) | 1.00 |  |  |  |  |  |  |  |  |  |  |  |
| Quartile 2 | 0.96 | 0.74 | 1.24 | 0.87 | 0.54 | 1.40 | 0.96 | 0.74 | 1.25 | 1.02 | 0.64 | 1.60 |
| Quartile 3 | 0.88 | 0.65 | 1.19 | 0.71 | 0.38 | 1.33 | 0.89 | 0.66 | 1.20 | 1.21 | 0.75 | 1.94 |
| Quartile 4 (highest) | 1.16 | 0.89 | 1.51 | 0.72 | 0.37 | 1.42 | 1.17 | 0.89 | 1.53 | 1.14 | 0.70 | 1.86 |
| *Social capital* | |  |  |  |  |  |  |  |  |  |  |  |
| Quartile 1 (lowest) | 1.00 |  |  |  |  |  |  |  |  |  |  |  |
| Quartile 2 | 1.25 | 0.96 | 1.64 | 1.27 | 0.97 | 1.66 | 1.28 | 0.68 | 2.44 | 1.58 | 0.85 | 2.93 |
| Quartile 3 | **1.62** | 1.27 | 2.06 | **1.65** | 1.29 | 2.10 | 1.58 | 0.95 | 2.62 | 1.53 | 0.93 | 2.52 |
| Quartile 4 (highest) | **1.91** | 1.44 | 2.52 | **1.92** | 1.45 | 2.53 | **2.45** | 1.42 | 4.22 | **2.61** | 1.52 | 4.47 |
| *Embodied cultural capital* | |  |  |  |  |  |  |  |  |  |  |  |
| Quartile 1 (lowest) | 1.00 |  |  |  |  |  |  |  |  |  |  |  |
| Quartile 2 | **1.43** | 1.11 | 1.84 | 1.31 | 0.84 | 2.04 | **1.54** | 1.00 | 2.36 | **1.43** | 1.11 | 1.85 |
| Quartile 3 | **1.72** | 1.33 | 2.23 | 1.15 | 0.68 | 1.95 | **1.94** | 1.25 | 3.01 | **1.75** | 1.34 | 2.27 |
| Quartile 4 (highest) | **3.15** | 2.31 | 4.29 | **2.73** | 1.35 | 5.52 | **2.70** | 1.54 | 4.75 | **3.23** | 2.36 | 4.41 |
| *Economic capital x Embodied cultural capital* | | | |  |  |  |  |  |  |  |  |  |
| Quartile 2 x Quartile 2 | | |  | 0.97 | 0.51 | 1.84 |  |  |  |  |  |  |
| Quartile 2 x Quartile 3 | | |  | 1.54 | 0.75 | 3.16 |  |  |  |  |  |  |
| Quartile 2 x Quartile 4 | | |  | 1.22 | 0.48 | 3.10 |  |  |  |  |  |  |
| Quartile 3 x Quartile 2 | | |  | 1.50 | 0.68 | 3.32 |  |  |  |  |  |  |
| Quartile 3 x Quartile 3 | | |  | 1.75 | 0.77 | 3.98 |  |  |  |  |  |  |
| Quartile 3 x Quartile 4 | | |  | 0.87 | 0.31 | 2.43 |  |  |  |  |  |  |
| Quartile 4 x Quartile 2 | | |  | 1.45 | 0.63 | 3.37 |  |  |  |  |  |  |
| Quartile 4 x Quartile 3 | | |  | 2.18 | 0.93 | 5.11 |  |  |  |  |  |  |
| Quartile 4 x Quartile 4 | | |  | 1.91 | 0.71 | 5.18 |  |  |  |  |  |  |
| *Social capital x Embodied cultural capital* | | |  |  |  |  |  |  |  |  |  |  |
| Quartile 2 x Quartile 2 | | |  |  |  |  | 0.90 | 0.41 | 2.00 |  |  |  |
| Quartile 2 x Quartile 3 | | |  |  |  |  | 0.82 | 0.36 | 1.87 |  |  |  |
| Quartile 2 x Quartile 4 | | |  |  |  |  | 1.47 | 0.58 | 3.78 |  |  |  |
| Quartile 3 x Quartile 2 | | |  |  |  |  | 1.00 | 0.52 | 1.93 |  |  |  |
| Quartile 3 x Quartile 3 | | |  |  |  |  | 0.93 | 0.49 | 1.79 |  |  |  |
| Quartile 3 x Quartile 4 | | |  |  |  |  | 1.33 | 0.60 | 2.97 |  |  |  |
| Quartile 4 x Quartile 2 | | |  |  |  |  | 0.72 | 0.34 | 1.55 |  |  |  |
| Quartile 4 x Quartile 3 | | |  |  |  |  | 0.70 | 0.34 | 1.47 |  |  |  |
| Quartile 4 x Quartile 4 | | |  |  |  |  | 0.82 | 0.34 | 2.00 |  |  |  |
| *Economic capital x Social capital* | | | |  |  |  |  |  |  |  |  |  |
| Quartile 2 x Quartile 2 | | |  |  |  |  |  |  |  | 0.65 | 0.28 | 1.50 |
| Quartile 2 x Quartile 3 | | |  |  |  |  |  |  |  | 0.63 | 0.26 | 1.51 |
| Quartile 2 x Quartile 4 | | |  |  |  |  |  |  |  | 0.98 | 0.43 | 2.22 |
| Quartile 3 x Quartile 2 | | |  |  |  |  |  |  |  | 1.35 | 0.69 | 2.62 |
| Quartile 3 x Quartile 3 | | |  |  |  |  |  |  |  | 0.65 | 0.30 | 1.39 |
| Quartile 3 x Quartile 4 | | |  |  |  |  |  |  |  | 1.18 | 0.59 | 2.36 |
| Quartile 4 x Quartile 2 | | |  |  |  |  |  |  |  | 0.69 | 0.33 | 1.46 |
| Quartile 4 x Quartile 3 | | |  |  |  |  |  |  |  | 0.51 | 0.22 | 1.20 |
| Quartile 4 x Quartile 4 | | |  |  |  |  |  |  |  | 0.76 | 0.35 | 1.67 |

For all three forms of capital, the reference category is quartile 1, the lowest quartile. Statistically significant estimates based on 95% confidence intervals are indicated in **bold**.

^*^The nested model ANOVA tests compared each model containing interaction terms with the main effects model. A model containing interaction terms can be said to explain more about the data than the main effects model if the ANOVA test p-value < 0.05. ANOVA test p-values < 0.05 are indicated in **bold**.

ANOVA: analysis of variance; CI: confidence interval.

*Vegetable consumption*

Table 4. Unadjusted regression model results: vegetable consumption

| Model | Main effects model  (Model 1) | | | Economic capital x Embodied cultural capital model  (Model 2A) | | | Social capital x Embodied cultural capital model  (Model 2B) | | | Economic capital x Social capital model  (Model 2C) | | |
| --- | --- | --- | --- | --- | --- | --- | --- | --- | --- | --- | --- | --- |
|  | Odds Ratios | 95% CI | | Odds Ratios | 95% CI | | Odds Ratios | 95% CI | | Odds Ratios | 95% CI | |
| Variable |  | Lower | Upper |  | Lower | Upper |  | Lower | Upper |  | Lower | Upper |
| *ANOVA test (p-value)^*^* |  |  |  | *0.597* |  |  | *0.183* |  |  | *0.816* |  |  |
| Intercept | **0.10** | 0.07 | **0.14** | **0.10** | 0.06 | 0.14 | **0.08** | 0.05 | 0.13 | **0.12** | 0.08 | 0.18 |
| *Economic capital* | |  |  |  |  |  |  |  |  |  |  |  |
| Quartile 1 (lowest) | 1.00 |  |  |  |  |  |  |  |  |  |  |  |
| Quartile 2 | 1.33 | 0.98 | 1.79 | 1.56 | 0.89 | 2.74 | 1.34 | 0.99 | 1.81 | 1.05 | 0.63 | 1.75 |
| Quartile 3 | **1.41** | 1.01 | 1.96 | 1.29 | 0.61 | 2.74 | **1.41** | 1.01 | 1.97 | 1.14 | 0.66 | 1.96 |
| Quartile 4 (highest) | 1.30 | 0.95 | 1.79 | 1.13 | 0.50 | 2.55 | 1.32 | 0.96 | 1.82 | 1.08 | 0.62 | 1.87 |
| *Social capital* | |  |  |  |  |  |  |  |  |  |  |  |
| Quartile 1 (lowest) | 1.00 |  |  |  |  |  |  |  |  |  |  |  |
| Quartile 2 | 1.33 | 0.99 | 1.79 | 1.33 | 0.99 | 1.79 | 1.64 | 0.78 | 3.44 | 1.17 | 0.58 | 2.36 |
| Quartile 3 | **1.33** | 1.01 | 1.75 | **1.35** | 1.03 | 1.78 | 1.56 | 0.80 | 3.05 | 1.13 | 0.62 | 2.04 |
| Quartile 4 (highest) | **1.61** | 1.19 | 2.19 | **1.63** | 1.20 | 2.21 | **2.70** | 1.33 | 5.47 | 0.91 | 0.45 | 1.85 |
| *Embodied cultural capital* | |  |  |  |  |  |  |  |  |  |  |  |
| Quartile 1 (lowest) | 1.00 |  |  |  |  |  |  |  |  |  |  |  |
| Quartile 2 | **1.66** | 1.23 | 2.25 | **1.85** | 1.05 | 3.24 | **1.98** | 1.16 | 3.37 | **1.65** | 1.22 | 2.24 |
| Quartile 3 | **2.32** | 1.72 | 3.14 | 1.75 | 0.89 | 3.47 | **3.46** | 2.05 | 5.86 | **2.33** | 1.72 | 3.15 |
| Quartile 4 (highest) | **3.11** | 2.19 | 4.43 | **4.79** | 2.20 | 10.42 | **2.64** | 1.31 | 5.32 | **3.09** | 2.17 | 4.41 |
| *Economic capital x Embodied cultural capital* | | | |  |  |  |  |  |  |  |  |  |
| Quartile 2 x Quartile 2 | | |  | 0.72 | 0.34 | 1.55 |  |  |  |  |  |  |
| Quartile 2 x Quartile 3 | | |  | 1.11 | 0.47 | 2.62 |  |  |  |  |  |  |
| Quartile 2 x Quartile 4 | | |  | 0.65 | 0.23 | 1.80 |  |  |  |  |  |  |
| Quartile 3 x Quartile 2 | | |  | 1.17 | 0.46 | 3.00 |  |  |  |  |  |  |
| Quartile 3 x Quartile 3 | | |  | 1.57 | 0.58 | 4.27 |  |  |  |  |  |  |
| Quartile 3 x Quartile 4 | | |  | 0.55 | 0.17 | 1.73 |  |  |  |  |  |  |
| Quartile 4 x Quartile 2 | | |  | 1.00 | 0.37 | 2.69 |  |  |  |  |  |  |
| Quartile 4 x Quartile 3 | | |  | 1.73 | 0.61 | 4.89 |  |  |  |  |  |  |
| Quartile 4 x Quartile 4 | | |  | 0.75 | 0.24 | 2.28 |  |  |  |  |  |  |
| *Social capital x Embodied cultural capital* | | |  |  |  |  |  |  |  |  |  |  |
| Quartile 2 x Quartile 2 | | |  |  |  |  | 0.93 | 0.38 | 2.29 |  |  |  |
| Quartile 2 x Quartile 3 | | |  |  |  |  | 0.46 | 0.18 | 1.16 |  |  |  |
| Quartile 2 x Quartile 4 | | |  |  |  |  | 1.46 | 0.50 | 4.22 |  |  |  |
| Quartile 3 x Quartile 2 | | |  |  |  |  | 0.77 | 0.34 | 1.76 |  |  |  |
| Quartile 3 x Quartile 3 | | |  |  |  |  | 0.74 | 0.33 | 1.67 |  |  |  |
| Quartile 3 x Quartile 4 | | |  |  |  |  | 1.21 | 0.44 | 3.32 |  |  |  |
| Quartile 4 x Quartile 2 | | |  |  |  |  | 0.60 | 0.25 | 1.45 |  |  |  |
| Quartile 4 x Quartile 3 | | |  |  |  |  | **0.40** | 0.16 | 0.96 |  |  |  |
| Quartile 4 x Quartile 4 | | |  |  |  |  | 0.86 | 0.30 | 2.43 |  |  |  |
| *Economic capital x Social capital* | | | |  |  |  |  |  |  |  |  |  |
| Quartile 2 x Quartile 2 | | |  |  |  |  |  |  |  | 1.15 | 0.48 | 2.77 |
| Quartile 2 x Quartile 3 | | |  |  |  |  |  |  |  | 1.17 | 0.45 | 3.04 |
| Quartile 2 x Quartile 4 | | |  |  |  |  |  |  |  | 1.19 | 0.48 | 2.97 |
| Quartile 3 x Quartile 2 | | |  |  |  |  |  |  |  | 1.24 | 0.57 | 2.70 |
| Quartile 3 x Quartile 3 | | |  |  |  |  |  |  |  | 1.23 | 0.53 | 2.82 |
| Quartile 3 x Quartile 4 | | |  |  |  |  |  |  |  | 1.21 | 0.56 | 2.63 |
| Quartile 4 x Quartile 2 | | |  |  |  |  |  |  |  | 2.30 | 0.93 | 5.69 |
| Quartile 4 x Quartile 3 | | |  |  |  |  |  |  |  | 2.03 | 0.76 | 5.44 |
| Quartile 4 x Quartile 4 | | |  |  |  |  |  |  |  | 1.77 | 0.70 | 4.46 |

For all three forms of capital, the reference category is quartile 1, the lowest quartile. Statistically significant estimates based on 95% confidence intervals are indicated in **bold**.

^*^The nested model ANOVA tests compared each model containing interaction terms with the main effects model. A model containing interaction terms can be said to explain more about the data than the main effects model if the ANOVA test p-value < 0.05. ANOVA test p-values < 0.05 are indicated in **bold**.

ANOVA: analysis of variance; CI: confidence interval.
