# Supplementary material for: Testing conditionality with Bourdieu's capital theory: How economic, social, and embodied cultural capital are associated with diet and physical activity in the Netherlands
Source: SSM Popul Health. 2023 Apr 10;22:101401. doi: 10.1016/j.ssmph.2023.101401 (PMC10139966; doi:10.1016/j.ssmph.2023.101401)
Supplement: Supplementary File 2 — Full regression model results for sports participation and leisure time walking or cycling [file mmc2.docx]

# Supplementary File 2: Full model results and ANOVA tests, physical activity

Table 1. Full regression model results: sports participation

| Model | Main effects model  (Model 1) | | | Economic capital x Embodied cultural capital model  (Model 2A) | | | Social capital x Embodied cultural capital model  (Model 2B) | | | Economic capital x Social capital model  (Model 2C) | | |
| --- | --- | --- | --- | --- | --- | --- | --- | --- | --- | --- | --- | --- |
|  | Odds Ratio | 95% CI | | Odds Ratio | 95% CI | | Odds Ratio | 95% CI | | Odds Ratio | 95% CI | |
| Variable |  | Lower | Upper |  | Lower | Upper |  | Lower | Upper |  | Lower | Upper |
| *ANOVA test (p-value)^*^* |  |  |  | *0.292* |  |  | *0.498* |  |  | ***0.042*** |  |  |
| Intercept | **1.82** | 1.07 | 3.10 | 1.74 | 0.97 | 3.11 | 1.72 | 0.97 | 3.08 | **2.59** | 1.43 | 4.72 |
| *Economic capital* | |  |  |  |  |  |  |  |  |  |  |  |
| Quartile 1 (lowest) | 1.00 |  |  |  |  |  |  |  |  |  |  |  |
| Quartile 2 | **1.72** | 1.32 | 2.23 | **1.97** | 1.28 | 3.03 | **1.74** | 1.33 | 2.26 | 1.02 | 0.62 | 1.67 |
| Quartile 3 | **2.09** | 1.53 | 2.86 | **2.51** | 1.40 | 4.49 | **2.11** | 1.54 | 2.89 | 1.44 | 0.84 | 2.47 |
| Quartile 4 (highest) | **2.36** | 1.75 | 3.17 | **1.80** | 1.00 | 3.26 | **2.37** | 1.76 | 3.19 | 1.56 | 0.90 | 2.70 |
| *Social capital* | |  |  |  |  |  |  |  |  |  |  |  |
| Quartile 1 (lowest) | 1.00 |  |  |  |  |  |  |  |  |  |  |  |
| Quartile 2 | 1.20 | 0.93 | 1.54 | 1.23 | 0.95 | 1.58 | 1.45 | 0.90 | 2.34 | 0.84 | 0.49 | 1.43 |
| Quartile 3 | **2.08** | 1.57 | 2.75 | **2.11** | 1.60 | 2.80 | **1.95** | 1.17 | 3.25 | 1.19 | 0.71 | 1.99 |
| Quartile 4 (highest) | **1.75** | 1.28 | 2.40 | **1.80** | 1.31 | 2.48 | **1.71** | 0.93 | 3.13 | 0.97 | 0.53 | 1.78 |
| *Embodied cultural capital* | |  |  |  |  |  |  |  |  |  |  |  |
| Quartile 1 (lowest) | 1.00 |  |  |  |  |  |  |  |  |  |  |  |
| Quartile 2 | **1.79** | 1.40 | 2.30 | **2.06** | 1.33 | 3.20 | **2.11** | 1.32 | 3.36 | **1.77** | 1.38 | 2.26 |
| Quartile 3 | **2.09** | 1.61 | 2.73 | **1.81** | 1.08 | 3.03 | **1.97** | 1.20 | 3.23 | **2.08** | 1.60 | 2.70 |
| Quartile 4 (highest) | **1.85** | 1.32 | 2.58 | **2.40** | 1.13 | 5.09 | 1.98 | 0.99 | 3.96 | **1.78** | 1.27 | 2.48 |
| *Economic capital x Embodied cultural capital* | | | |  |  |  |  |  |  |  |  |  |
| Quartile 2 x Quartile 2 | | |  | 0.67 | 0.37 | 1.24 |  |  |  |  |  |  |
| Quartile 2 x Quartile 3 | | |  | 1.06 | 0.54 | 2.12 |  |  |  |  |  |  |
| Quartile 2 x Quartile 4 | | |  | 0.85 | 0.32 | 2.24 |  |  |  |  |  |  |
| Quartile 3 x Quartile 2 | | |  | 0.90 | 0.41 | 1.98 |  |  |  |  |  |  |
| Quartile 3 x Quartile 3 | | |  | 1.05 | 0.46 | 2.41 |  |  |  |  |  |  |
| Quartile 3 x Quartile 4 | | |  | 0.40 | 0.14 | 1.17 |  |  |  |  |  |  |
| Quartile 4 x Quartile 2 | | |  | 1.16 | 0.53 | 2.54 |  |  |  |  |  |  |
| Quartile 4 x Quartile 3 | | |  | 1.66 | 0.75 | 3.70 |  |  |  |  |  |  |
| Quartile 4 x Quartile 4 | | |  | 1.17 | 0.42 | 3.22 |  |  |  |  |  |  |
| *Social capital x Embodied cultural capital* | | |  |  |  |  |  |  |  |  |  |  |
| Quartile 2 x Quartile 2 | | |  |  |  |  | 0.69 | 0.35 | 1.32 |  |  |  |
| Quartile 2 x Quartile 3 | | |  |  |  |  | 0.88 | 0.45 | 1.70 |  |  |  |
| Quartile 2 x Quartile 4 | | |  |  |  |  | 0.79 | 0.32 | 1.91 |  |  |  |
| Quartile 3 x Quartile 2 | | |  |  |  |  | 1.07 | 0.50 | 2.27 |  |  |  |
| Quartile 3 x Quartile 3 | | |  |  |  |  | 1.10 | 0.53 | 2.32 |  |  |  |
| Quartile 3 x Quartile 4 | | |  |  |  |  | 1.11 | 0.42 | 2.94 |  |  |  |
| Quartile 4 x Quartile 2 | | |  |  |  |  | 0.70 | 0.31 | 1.60 |  |  |  |
| Quartile 4 x Quartile 3 | | |  |  |  |  | 1.84 | 0.76 | 4.44 |  |  |  |
| Quartile 4 x Quartile 4 | | |  |  |  |  | 0.90 | 0.31 | 2.61 |  |  |  |
| *Economic capital x Social capital* | | | |  |  |  |  |  |  |  |  |  |
| Quartile 2 x Quartile 2 | | |  |  |  |  |  |  |  | 1.93 | 0.96 | 3.89 |
| Quartile 2 x Quartile 3 | | |  |  |  |  |  |  |  | **2.46** | 1.18 | 5.12 |
| Quartile 2 x Quartile 4 | | |  |  |  |  |  |  |  | 1.93 | 0.87 | 4.29 |
| Quartile 3 x Quartile 2 | | |  |  |  |  |  |  |  | 1.65 | 0.75 | 3.61 |
| Quartile 3 x Quartile 3 | | |  |  |  |  |  |  |  | 1.32 | 0.57 | 3.06 |
| Quartile 3 x Quartile 4 | | |  |  |  |  |  |  |  | **3.07** | 1.11 | 8.50 |
| Quartile 4 x Quartile 2 | | |  |  |  |  |  |  |  | 1.33 | 0.63 | 2.81 |
| Quartile 4 x Quartile 3 | | |  |  |  |  |  |  |  | **2.71** | 1.22 | 6.05 |
| Quartile 4 x Quartile 4 | | |  |  |  |  |  |  |  | 2.43 | 0.97 | 6.11 |
| *Demographic control variables* | | | |  |  |  |  |  |  |  |  |  |
| Age | **0.97** | 0.96 | 0.98 | **0.97** | 0.96 | 0.98 | **0.97** | 0.96 | 0.98 | **0.97** | 0.96 | 0.98 |
| Female | **0.64** | 0.52 | 0.79 | **0.64** | 0.52 | 0.79 | **0.64** | 0.52 | 0.80 | **0.64** | 0.52 | 0.79 |
| Country of birth: NL | 1.21 | 0.89 | 1.65 | 1.21 | 0.89 | 1.65 | 1.21 | 0.88 | 1.64 | 1.20 | 0.88 | 1.63 |
| Work: Homemaker | 1.07 | 0.66 | 1.73 | 1.05 | 0.65 | 1.70 | 1.08 | 0.67 | 1.75 | 1.06 | 0.66 | 1.72 |
| Work: Other | 1.56 | 0.88 | 2.79 | 1.55 | 0.87 | 2.77 | 1.57 | 0.87 | 2.81 | 1.61 | 0.91 | 2.85 |
| Work: Part time | 1.10 | 0.84 | 1.44 | 1.12 | 0.86 | 1.47 | 1.09 | 0.84 | 1.42 | 1.08 | 0.83 | 1.41 |
| Work: Retired | 0.97 | 0.67 | 1.41 | 0.98 | 0.68 | 1.42 | 0.98 | 0.67 | 1.41 | 0.97 | 0.67 | 1.40 |
| Work: Unemployed | 0.78 | 0.52 | 1.15 | 0.79 | 0.53 | 1.17 | 0.78 | 0.52 | 1.15 | 0.78 | 0.53 | 1.15 |

For all three forms of capital, the reference category is quartile 1, the lowest quartile. Statistically significant estimates based on 95% confidence intervals are indicated in **bold**.

^*^The nested model ANOVA tests compared each model containing interaction terms with the main effects model. A model containing interaction terms can be said to explain more about the data than the main effects model if the ANOVA test p-value < 0.05. ANOVA test p-values < 0.05 are indicated in **bold**.

ANOVA: analysis of variance; CI: confidence interval.

Table 2. Full regression model results: leisure time walking or cycling

| Model | Main effects model  (Model 1) | | | Economic capital x Embodied cultural capital model  (Model 2A) | | | Social capital x Embodied cultural capital model  (Model 2B) | | | Economic capital x Social capital model  (Model 2C) | | |
| --- | --- | --- | --- | --- | --- | --- | --- | --- | --- | --- | --- | --- |
|  | Odds Ratio | 95% CI | | Odds Ratio | 95% CI | | Odds Ratio | 95% CI | | Odds Ratio | 95% CI | |
| Variable |  | Lower | Upper |  | Lower | Upper |  | Lower | Upper |  | Lower | Upper |
| *ANOVA test (p-value)^*^* |  |  |  | *0.474* |  |  | *0.199* |  |  | *0.820* |  |  |
| Intercept | **0.13** | 0.08 | 0.22 | **0.13** | 0.07 | 0.22 | **0.13** | 0.08 | 0.23 | **0.16** | 0.09 | 0.28 |
| *Economic capital* | |  |  |  |  |  |  |  |  |  |  |  |
| Quartile 1 (lowest) | 1.00 |  |  |  |  |  |  |  |  |  |  |  |
| Quartile 2 | 0.91 | 0.71 | 1.18 | 1.03 | 0.68 | 1.54 | 0.90 | 0.69 | 1.16 | 0.73 | 0.46 | 1.15 |
| Quartile 3 | 0.92 | 0.67 | 1.26 | 0.71 | 0.39 | 1.30 | 0.90 | 0.66 | 1.23 | 0.72 | 0.43 | 1.20 |
| Quartile 4 (highest) | 1.17 | 0.86 | 1.57 | 1.04 | 0.57 | 1.91 | 1.16 | 0.86 | 1.57 | 0.91 | 0.53 | 1.56 |
| *Social capital* | |  |  |  |  |  |  |  |  |  |  |  |
| Quartile 1 (lowest) | 1.00 |  |  |  |  |  |  |  |  |  |  |  |
| Quartile 2 | 1.15 | 0.90 | 1.46 | 1.14 | 0.90 | 1.46 | 1.11 | 0.70 | 1.77 | 0.86 | 0.52 | 1.43 |
| Quartile 3 | 1.27 | 0.98 | 1.65 | 1.26 | 0.97 | 1.65 | **1.73** | 1.07 | 2.80 | 1.14 | 0.66 | 1.97 |
| Quartile 4 (highest) | 0.97 | 0.71 | 1.32 | 0.97 | 0.71 | 1.32 | 0.56 | 0.31 | 1.00 | 0.63 | 0.35 | 1.12 |
| *Embodied cultural capital* | |  |  |  |  |  |  |  |  |  |  |  |
| Quartile 1 (lowest) | 1.00 |  |  |  |  |  |  |  |  |  |  |  |
| Quartile 2 | **1.57** | 1.24 | 2.00 | **1.62** | 1.05 | 2.50 | **1.57** | 1.02 | 2.40 | **1.56** | 1.23 | 1.99 |
| Quartile 3 | **2.03** | 1.56 | 2.62 | **1.90** | 1.09 | 3.32 | **1.97** | 1.24 | 3.13 | **2.02** | 1.56 | 2.62 |
| Quartile 4 (highest) | **3.42** | 2.37 | 4.96 | **3.07** | 1.39 | 6.78 | **3.12** | 1.51 | 6.48 | **3.37** | 2.33 | 4.87 |
| *Economic capital x Embodied cultural capital* | | | |  |  |  |  |  |  |  |  |  |
| Quartile 2 x Quartile 2 | | |  | 0.69 | 0.38 | 1.27 |  |  |  |  |  |  |
| Quartile 2 x Quartile 3 | | |  | 1.06 | 0.53 | 2.15 |  |  |  |  |  |  |
| Quartile 2 x Quartile 4 | | |  | 1.19 | 0.41 | 3.42 |  |  |  |  |  |  |
| Quartile 3 x Quartile 2 | | |  | 1.53 | 0.70 | 3.37 |  |  |  |  |  |  |
| Quartile 3 x Quartile 3 | | |  | 1.26 | 0.55 | 2.92 |  |  |  |  |  |  |
| Quartile 3 x Quartile 4 | | |  | 1.54 | 0.46 | 5.12 |  |  |  |  |  |  |
| Quartile 4 x Quartile 2 | | |  | 1.27 | 0.59 | 2.70 |  |  |  |  |  |  |
| Quartile 4 x Quartile 3 | | |  | 1.14 | 0.50 | 2.63 |  |  |  |  |  |  |
| Quartile 4 x Quartile 4 | | |  | 1.08 | 0.36 | 3.19 |  |  |  |  |  |  |
| *Social capital x Embodied cultural capital* | | |  |  |  |  |  |  |  |  |  |  |
| Quartile 2 x Quartile 2 | | |  |  |  |  | 1.11 | 0.60 | 2.05 |  |  |  |
| Quartile 2 x Quartile 3 | | |  |  |  |  | 0.98 | 0.51 | 1.89 |  |  |  |
| Quartile 2 x Quartile 4 | | |  |  |  |  | 1.05 | 0.40 | 2.79 |  |  |  |
| Quartile 3 x Quartile 2 | | |  |  |  |  | 0.57 | 0.29 | 1.11 |  |  |  |
| Quartile 3 x Quartile 3 | | |  |  |  |  | 0.73 | 0.36 | 1.46 |  |  |  |
| Quartile 3 x Quartile 4 | | |  |  |  |  | 0.88 | 0.31 | 2.50 |  |  |  |
| Quartile 4 x Quartile 2 | | |  |  |  |  | 2.04 | 0.90 | 4.64 |  |  |  |
| Quartile 4 x Quartile 3 | | |  |  |  |  | 2.20 | 0.98 | 4.95 |  |  |  |
| Quartile 4 x Quartile 4 | | |  |  |  |  | 2.07 | 0.63 | 6.82 |  |  |  |
| *Economic capital x Social capital* | | | |  |  |  |  |  |  |  |  |  |
| Quartile 2 x Quartile 2 | | |  |  |  |  |  |  |  | 1.41 | 0.72 | 2.76 |
| Quartile 2 x Quartile 3 | | |  |  |  |  |  |  |  | 1.18 | 0.55 | 2.52 |
| Quartile 2 x Quartile 4 | | |  |  |  |  |  |  |  | 1.79 | 0.80 | 4.03 |
| Quartile 3 x Quartile 2 | | |  |  |  |  |  |  |  | 1.50 | 0.72 | 3.15 |
| Quartile 3 x Quartile 3 | | |  |  |  |  |  |  |  | 1.11 | 0.50 | 2.48 |
| Quartile 3 x Quartile 4 | | |  |  |  |  |  |  |  | 1.90 | 0.78 | 4.61 |
| Quartile 4 x Quartile 2 | | |  |  |  |  |  |  |  | 1.48 | 0.71 | 3.09 |
| Quartile 4 x Quartile 3 | | |  |  |  |  |  |  |  | 1.18 | 0.53 | 2.61 |
| Quartile 4 x Quartile 4 | | |  |  |  |  |  |  |  | 1.83 | 0.73 | 4.59 |
| *Demographic control variables* | | | |  |  |  |  |  |  |  |  |  |
| Age | **1.03** | 1.02 | 1.04 | **1.03** | 1.03 | 1.04 | **1.03** | 1.03 | 1.04 | **1.03** | 1.03 | 1.04 |
| Female | 0.92 | 0.75 | 1.13 | 0.93 | 0.75 | 1.14 | 0.92 | 0.75 | 1.13 | 0.92 | 0.75 | 1.14 |
| Country of birth: NL | **1.32** | 1.00 | 1.74 | **1.33** | 1.01 | 1.75 | **1.32** | 1.00 | 1.74 | 1.31 | 0.99 | 1.72 |
| Work: Homemaker | **3.32** | 1.85 | 5.96 | **3.22** | 1.80 | 5.77 | **3.29** | 1.81 | 5.96 | **3.37** | 1.86 | 6.09 |
| Work: Other | 1.57 | 0.96 | 2.57 | 1.58 | 0.96 | 2.59 | 1.60 | 0.98 | 2.63 | 1.59 | 0.97 | 2.60 |
| Work: Part time | 1.21 | 0.95 | 1.55 | 1.22 | 0.95 | 1.55 | 1.21 | 0.95 | 1.55 | 1.21 | 0.95 | 1.54 |
| Work: Retired | **2.34** | 1.58 | 3.48 | **2.31** | 1.56 | 3.42 | **2.33** | 1.57 | 3.47 | **2.35** | 1.58 | 3.50 |
| Work: Unemployed | **1.53** | 1.06 | 2.22 | **1.53** | 1.06 | 2.21 | **1.56** | 1.08 | 2.25 | **1.53** | 1.06 | 2.21 |

For all three forms of capital, the reference category is quartile 1, the lowest quartile. Statistically significant estimates based on 95% confidence intervals are indicated in **bold**.

^*^The nested model ANOVA tests compared each model containing interaction terms with the main effects model. A model containing interaction terms can be said to explain more about the data than the main effects model if the ANOVA test p-value < 0.05. ANOVA test p-values < 0.05 are indicated in **bold**.

ANOVA: analysis of variance; CI: confidence interval.
