# Supplementary material for: Testing conditionality with Bourdieu's capital theory: How economic, social, and embodied cultural capital are associated with diet and physical activity in the Netherlands
Source: SSM Popul Health. 2023 Apr 10;22:101401. doi: 10.1016/j.ssmph.2023.101401 (PMC10139966; doi:10.1016/j.ssmph.2023.101401)
Supplement: Supplementary File 3 — Full regression model results for fruit consumption and vegetable consumption [file mmc3.docx]

# Supplementary File 3: Full model results and ANOVA tests, healthy diet

Table 1. Full regression model results: fruit consumption

| Model | Main effects model  (Model 1) | | | Economic capital x Embodied cultural capital model  (Model 2A) | | | Social capital x Embodied cultural capital model  (Model 2B) | | | Economic capital x Social capital model  (Model 2C) | | |
| --- | --- | --- | --- | --- | --- | --- | --- | --- | --- | --- | --- | --- |
|  | Odds Ratios | 95% CI | | Odds Ratios | 95% CI | | Odds Ratios | 95% CI | | Odds Ratios | 95% CI | |
| Variable |  | Lower | Upper |  | Lower | Upper |  | Lower | Upper |  | Lower | Upper |
| *ANOVA test (p-value)^*^* |  |  |  | *0.524* |  |  | *0.952* |  |  | *0.305* |  |  |
| Intercept | **0.09** | 0.05 | 0.16 | **0.10** | 0.06 | 0.18 | **0.09** | 0.05 | 0.17 | **0.08** | 0.05 | 0.15 |
| *Economic capital* |  |  |  |  |  |  |  |  |  |  |  |  |
| Quartile 1 (lowest) | 1.00 |  |  |  |  |  |  |  |  |  |  |  |
| Quartile 2 | 1.03 | 0.79 | 1.35 | 0.98 | 0.60 | 1.58 | 1.03 | 0.79 | 1.36 | 1.11 | 0.69 | 1.77 |
| Quartile 3 | 0.97 | 0.71 | 1.32 | 0.83 | 0.44 | 1.58 | 0.97 | 0.71 | 1.33 | 1.34 | 0.83 | 2.19 |
| Quartile 4 (highest) | 1.24 | 0.93 | 1.66 | 0.84 | 0.42 | 1.71 | 1.25 | 0.93 | 1.67 | 1.23 | 0.74 | 2.03 |
| *Social capital* |  |  |  |  |  |  |  |  |  |  |  |  |
| Quartile 1 (lowest) | 1.00 |  |  |  |  |  |  |  |  |  |  |  |
| Quartile 2 | 1.29 | 0.98 | 1.70 | 1.30 | 0.99 | 1.71 | 1.26 | 0.66 | 2.40 | 1.67 | 0.90 | 3.12 |
| Quartile 3 | **1.57** | 1.23 | 2.01 | **1.60** | 1.25 | 2.04 | 1.45 | 0.87 | 2.43 | 1.51 | 0.92 | 2.50 |
| Quartile 4 (highest) | **1.82** | 1.37 | 2.43 | **1.83** | 1.37 | 2.44 | **2.16** | 1.24 | 3.78 | **2.43** | 1.40 | 4.24 |
| *Embodied cultural capital* | | | | | | | | | | | | |
| Quartile 1 (lowest) | 1.00 |  |  |  |  |  |  |  |  |  |  |  |
| Quartile 2 | **1.43** | 1.10 | 1.86 | 1.31 | 0.84 | 2.05 | 1.46 | 0.95 | 2.26 | **1.44** | 1.11 | 1.87 |
| Quartile 3 | **1.67** | 1.28 | 2.18 | 1.21 | 0.71 | 2.07 | **1.77** | 1.13 | 2.76 | **1.70** | 1.30 | 2.22 |
| Quartile 4 (highest) | **2.61** | 1.90 | 3.58 | **2.74** | 1.31 | 5.75 | **2.26** | 1.27 | 4.02 | **2.68** | 1.95 | 3.68 |
| *Economic capital x Embodied cultural capital* | | | | | | | | | | | | |
| Quartile 2 x Quartile 2 |  |  |  | 0.94 | 0.49 | 1.80 |  |  |  |  |  |  |
| Quartile 2 x Quartile 3 |  |  |  | 1.44 | 0.70 | 2.97 |  |  |  |  |  |  |
| Quartile 2 x Quartile 4 |  |  |  | 1.02 | 0.39 | 2.68 |  |  |  |  |  |  |
| Quartile 3 x Quartile 2 |  |  |  | 1.44 | 0.64 | 3.21 |  |  |  |  |  |  |
| Quartile 3 x Quartile 3 |  |  |  | 1.54 | 0.66 | 3.56 |  |  |  |  |  |  |
| Quartile 3 x Quartile 4 |  |  |  | 0.70 | 0.24 | 2.02 |  |  |  |  |  |  |
| Quartile 4 x Quartile 2 |  |  |  | 1.51 | 0.64 | 3.58 |  |  |  |  |  |  |
| Quartile 4 x Quartile 3 |  |  |  | 1.85 | 0.77 | 4.45 |  |  |  |  |  |  |
| Quartile 4 x Quartile 4 |  |  |  | 1.42 | 0.50 | 4.02 |  |  |  |  |  |  |
| *Social capital x Embodied cultural capital* | |  |  |  |  |  |  |  |  |  |  |  |
| Quartile 2 x Quartile 2 |  |  |  |  |  |  | 1.00 | 0.44 | 2.24 |  |  |  |
| Quartile 2 x Quartile 3 |  |  |  |  |  |  | 0.91 | 0.40 | 2.08 |  |  |  |
| Quartile 2 x Quartile 4 |  |  |  |  |  |  | 1.38 | 0.53 | 3.57 |  |  |  |
| Quartile 3 x Quartile 2 |  |  |  |  |  |  | 1.12 | 0.58 | 2.17 |  |  |  |
| Quartile 3 x Quartile 3 |  |  |  |  |  |  | 1.01 | 0.52 | 1.95 |  |  |  |
| Quartile 3 x Quartile 4 |  |  |  |  |  |  | 1.31 | 0.58 | 2.94 |  |  |  |
| Quartile 4 x Quartile 2 |  |  |  |  |  |  | 0.74 | 0.34 | 1.60 |  |  |  |
| Quartile 4 x Quartile 3 |  |  |  |  |  |  | 0.81 | 0.38 | 1.73 |  |  |  |
| Quartile 4 x Quartile 4 |  |  |  |  |  |  | 0.94 | 0.37 | 2.34 |  |  |  |
| *Economic capital x Social capital* | |  |  |  |  |  |  |  |  |  |  |  |
| Quartile 2 x Quartile 2 |  |  |  |  |  |  |  |  |  | 0.63 | 0.27 | 1.47 |
| Quartile 2 x Quartile 3 |  |  |  |  |  |  |  |  |  | 1.32 | 0.67 | 2.58 |
| Quartile 2 x Quartile 4 |  |  |  |  |  |  |  |  |  | 0.70 | 0.33 | 1.51 |
| Quartile 3 x Quartile 2 |  |  |  |  |  |  |  |  |  | 0.59 | 0.24 | 1.44 |
| Quartile 3 x Quartile 3 |  |  |  |  |  |  |  |  |  | 0.63 | 0.29 | 1.36 |
| Quartile 3 x Quartile 4 |  |  |  |  |  |  |  |  |  | 0.52 | 0.22 | 1.25 |
| Quartile 4 x Quartile 2 |  |  |  |  |  |  |  |  |  | 0.96 | 0.42 | 2.21 |
| Quartile 4 x Quartile 3 |  |  |  |  |  |  |  |  |  | 1.16 | 0.58 | 2.33 |
| Quartile 4 x Quartile 4 |  |  |  |  |  |  |  |  |  | 0.80 | 0.36 | 1.79 |
| *Demographic control variables* | |  |  |  |  |  |  |  |  |  |  |  |
| Age | **1.02** | 1.01 | 1.03 | **1.02** | 1.01 | 1.03 | **1.02** | 1.01 | 1.03 | **1.02** | 1.01 | 1.03 |
| Female | **1.84** | 1.50 | 2.26 | **1.84** | 1.49 | 2.26 | **1.83** | 1.49 | 2.25 | **1.84** | 1.50 | 2.27 |
| Country of birth: NL | **0.73** | 0.55 | 0.98 | **0.73** | 0.55 | 0.98 | **0.73** | 0.55 | 0.97 | **0.74** | 0.55 | 0.99 |
| Work: Homemaker | 0.82 | 0.51 | 1.30 | 0.81 | 0.51 | 1.29 | 0.82 | 0.52 | 1.31 | 0.82 | 0.52 | 1.31 |
| Work: Other | **1.32** | 0.78 | 2.25 | 1.32 | 0.78 | 2.25 | 1.31 | 0.77 | 2.24 | 1.35 | 0.79 | 2.32 |
| Work: Part time | 0.79 | 0.61 | 1.03 | 0.80 | 0.62 | 1.04 | 0.80 | 0.62 | 1.03 | 0.79 | 0.61 | 1.03 |
| Work: Retired | 0.86 | 0.60 | 1.24 | 0.87 | 0.61 | 1.24 | 0.87 | 0.61 | 1.24 | 0.87 | 0.61 | 1.24 |
| Work: Unemployed | **0.57** | 0.38 | 0.86 | **0.58** | 0.38 | 0.87 | **0.57** | 0.38 | 0.86 | **0.57** | 0.38 | 0.86 |

For all three forms of capital, the reference category is quartile 1, the lowest quartile. Statistically significant estimates based on 95% confidence intervals are indicated in **bold**.

^*^The nested model ANOVA tests compared each model containing interaction terms with the main effects model. A model containing interaction terms can be said to explain more about the data than the main effects model if the ANOVA test p-value < 0.05. ANOVA test p-values < 0.05 are indicated in **bold**.

ANOVA: analysis of variance; CI: confidence interval.

Table 2. Full regression model results: vegetable consumption

| Model | Main effects model  (Model 1) | | | Economic capital x Embodied cultural capital model  (Model 2A) | | | Social capital x Embodied cultural capital model  (Model 2B) | | | Economic capital x Social capital model  (Model 2C) | | |
| --- | --- | --- | --- | --- | --- | --- | --- | --- | --- | --- | --- | --- |
|  | Odds Ratios | 95% CI | | Odds Ratios | 95% CI | | Odds Ratios | 95% CI | | Odds Ratios | 95% CI | |
| Variable |  | Lower | Upper |  | Lower | Upper |  | Lower | Upper |  | Lower | Upper |
| *ANOVA test (p-value)^*^* |  |  |  | *0.640* |  |  | *0.169* |  |  | *0.848* |  |  |
| Intercept | **0.17** | 0.10 | 0.31 | **0.18** | 0.09 | 0.34 | **0.15** | 0.08 | 0.30 | **0.21** | 0.11 | 0.39 |
| *Economic capital* |  |  |  |  |  |  |  |  |  |  |  |  |
| Quartile 1 (lowest) | 1.00 |  |  |  |  |  |  |  |  |  |  |  |
| Quartile 2 | 1.36 | 0.99 | 1.86 | 1.51 | 0.85 | 2.66 | 1.35 | 0.99 | 1.86 | 1.08 | 0.63 | 1.84 |
| Quartile 3 | **1.49** | 1.04 | 2.12 | 1.29 | 0.60 | 2.76 | **1.48** | 1.04 | 2.10 | 1.21 | 0.69 | 2.13 |
| Quartile 4 (highest) | **1.41** | 1.00 | 1.98 | 1.15 | 0.50 | 2.65 | **1.41** | 1.00 | 1.99 | 1.17 | 0.65 | 2.08 |
| *Social capital* |  |  |  |  |  |  |  |  |  |  |  |  |
| Quartile 1 (lowest) | 1.00 |  |  |  |  |  |  |  |  |  |  |  |
| Quartile 2 | 1.33 | 0.99 | 1.80 | 1.33 | 0.99 | 1.80 | 1.74 | 0.82 | 3.66 | 1.24 | 0.61 | 2.55 |
| Quartile 3 | 1.30 | 0.99 | 1.72 | **1.32** | 1.00 | 1.74 | 1.53 | 0.77 | 3.03 | 1.08 | 0.58 | 2.02 |
| Quartile 4 (highest) | **1.54** | 1.13 | 2.10 | **1.56** | 1.14 | 2.13 | **2.59** | 1.29 | 5.19 | 0.86 | 0.41 | 1.81 |
| *Embodied cultural capital* | | | | | | | | | | | | |
| Quartile 1 (lowest) | 1.00 |  |  |  |  |  |  |  |  |  |  |  |
| Quartile 2 | **1.56** | 1.15 | 2.13 | 1.67 | 0.93 | 2.97 | **1.90** | 1.11 | 3.26 | **1.56** | 1.14 | 2.12 |
| Quartile 3 | **2.22** | 1.64 | 3.01 | 1.58 | 0.78 | 3.19 | **3.38** | 2.00 | 5.71 | **2.22** | 1.64 | 3.01 |
| Quartile 4 (highest) | **3.01** | 2.11 | 4.30 | **4.40** | 1.95 | 9.94 | **2.54** | 1.25 | 5.14 | **2.99** | 2.09 | 4.27 |
| *Economic capital x Embodied cultural capital* | | | | | | | | | | | | |
| Quartile 2 x Quartile 2 |  |  |  | 0.77 | 0.35 | 1.68 |  |  |  |  |  |  |
| Quartile 2 x Quartile 3 |  |  |  | 1.20 | 0.50 | 2.87 |  |  |  |  |  |  |
| Quartile 2 x Quartile 4 |  |  |  | 0.70 | 0.25 | 1.99 |  |  |  |  |  |  |
| Quartile 3 x Quartile 2 |  |  |  | 1.21 | 0.47 | 3.14 |  |  |  |  |  |  |
| Quartile 3 x Quartile 3 |  |  |  | 1.74 | 0.63 | 4.80 |  |  |  |  |  |  |
| Quartile 3 x Quartile 4 |  |  |  | 0.60 | 0.19 | 1.96 |  |  |  |  |  |  |
| Quartile 4 x Quartile 2 |  |  |  | 1.07 | 0.39 | 2.93 |  |  |  |  |  |  |
| Quartile 4 x Quartile 3 |  |  |  | 1.87 | 0.65 | 5.40 |  |  |  |  |  |  |
| Quartile 4 x Quartile 4 |  |  |  | 0.79 | 0.25 | 2.52 |  |  |  |  |  |  |
| *Social capital x Embodied cultural capital* | |  |  |  |  |  |  |  |  |  |  |  |
| Quartile 2 x Quartile 2 |  |  |  |  |  |  | 0.87 | 0.35 | 2.14 |  |  |  |
| Quartile 2 x Quartile 3 |  |  |  |  |  |  | 0.44 | 0.17 | 1.10 |  |  |  |
| Quartile 2 x Quartile 4 |  |  |  |  |  |  | 1.37 | 0.47 | 3.99 |  |  |  |
| Quartile 3 x Quartile 2 |  |  |  |  |  |  | 0.76 | 0.33 | 1.76 |  |  |  |
| Quartile 3 x Quartile 3 |  |  |  |  |  |  | 0.74 | 0.32 | 1.67 |  |  |  |
| Quartile 3 x Quartile 4 |  |  |  |  |  |  | 1.24 | 0.45 | 3.45 |  |  |  |
| Quartile 4 x Quartile 2 |  |  |  |  |  |  | 0.57 | 0.23 | 1.38 |  |  |  |
| Quartile 4 x Quartile 3 |  |  |  |  |  |  | **0.38** | 0.16 | 0.93 |  |  |  |
| Quartile 4 x Quartile 4 |  |  |  |  |  |  | 0.95 | 0.34 | 2.70 |  |  |  |
| *Economic capital x Social capital* | |  |  |  |  |  |  |  |  |  |  |  |
| Quartile 2 x Quartile 2 |  |  |  |  |  |  |  |  |  | 1.09 | 0.45 | 2.65 |
| Quartile 2 x Quartile 3 |  |  |  |  |  |  |  |  |  | 1.29 | 0.57 | 2.90 |
| Quartile 2 x Quartile 4 |  |  |  |  |  |  |  |  |  | 2.20 | 0.89 | 5.46 |
| Quartile 3 x Quartile 2 |  |  |  |  |  |  |  |  |  | 1.10 | 0.42 | 2.84 |
| Quartile 3 x Quartile 3 |  |  |  |  |  |  |  |  |  | 1.24 | 0.53 | 2.91 |
| Quartile 3 x Quartile 4 |  |  |  |  |  |  |  |  |  | 2.08 | 0.76 | 5.70 |
| Quartile 4 x Quartile 2 |  |  |  |  |  |  |  |  |  | 1.11 | 0.44 | 2.81 |
| Quartile 4 x Quartile 3 |  |  |  |  |  |  |  |  |  | 1.23 | 0.55 | 2.72 |
| Quartile 4 x Quartile 4 |  |  |  |  |  |  |  |  |  | 1.84 | 0.71 | 4.76 |
| *Demographic control variables* | |  |  |  |  |  |  |  |  |  |  |  |
| Age | 0.99 | 0.98 | 1.00 | 0.99 | 0.98 | 1.00 | 0.99 | 0.98 | 1.00 | 0.99 | 0.98 | 1.00 |
| Female | **1.40** | 1.11 | 1.76 | **1.40** | 1.11 | 1.76 | **1.37** | 1.09 | 1.73 | **1.39** | 1.11 | 1.75 |
| Country of birth: NL | **0.70** | 0.50 | 0.97 | **0.70** | 0.50 | 0.97 | **0.70** | 0.50 | 0.96 | **0.69** | 0.50 | 0.96 |
| Work: Homemaker | 1.15 | 0.66 | 2.00 | 1.14 | 0.66 | 1.99 | 1.18 | 0.68 | 2.05 | 1.17 | 0.68 | 2.04 |
| Work: Other | **2.45** | 1.46 | 4.13 | **2.42** | 1.44 | 4.07 | **2.41** | 1.44 | 4.04 | **2.45** | 1.46 | 4.13 |
| Work: Part time | 0.87 | 0.66 | 1.15 | 0.88 | 0.66 | 1.16 | 0.88 | 0.67 | 1.16 | 0.87 | 0.66 | 1.15 |
| Work: Retired | 1.13 | 0.75 | 1.71 | 1.14 | 0.75 | 1.72 | 1.15 | 0.76 | 1.73 | 1.13 | 0.75 | 1.71 |
| Work: Unemployed | **0.58** | 0.36 | 0.95 | **0.58** | 0.36 | 0.95 | **0.57** | 0.35 | 0.92 | **0.58** | 0.36 | 0.95 |

For all three forms of capital, the reference category is quartile 1, the lowest quartile. Statistically significant estimates based on 95% confidence intervals are indicated in **bold**.

^*^The nested model ANOVA tests compared each model containing interaction terms with the main effects model. A model containing interaction terms can be said to explain more about the data than the main effects model if the ANOVA test p-value < 0.05. ANOVA test p-values < 0.05 are indicated in **bold**.

ANOVA: analysis of variance; CI: confidence interval.
